# Digital mammographic density and breast cancer risk: a case–control study of six alternative density assessment methods

**DOI:** 10.1186/s13058-014-0439-1

**Published:** 2014-09-20

**Authors:** Amanda Eng, Zoe Gallant, John Shepherd, Valerie McCormack, Jingmei Li, Mitch Dowsett, Sarah Vinnicombe, Steve Allen, Isabel dos-Santos-Silva

**Affiliations:** 1 Department of Non-Communicable Disease Epidemiology, London School of Hygiene and Tropical Medicine, London, UK; 2 Centre for Public Health Research, Massey University, Wellington, New Zealand; 3 Radiology and Biomedical Imaging, University of California, San Francisco, USA; 4 Section of Environment and Radiation, International Agency for Research on Cancer, Lyon, France; 5 Human Genetics, Genome Institute of Singapore, Singapore, Singapore; 6 Academic Biochemistry, Royal Marsden Hospital, London, UK; 7 Division of Imaging and Technology, Ninewells Hospital Medical School, University of Dundee, Dundee, UK; 8 Director of the Central and East London Breast Screening Service, CELBSS, Dundee, UK; 9 Department of Imaging, Royal Marsden NHS Foundation Trust, London, UK

## Abstract

**Introduction:**

Mammographic density is a strong breast cancer risk factor and a major determinant of screening sensitivity. However, there is currently no validated estimation method for full-field digital mammography (FFDM).

**Methods:**

The performance of three area-based approaches (BI-RADS, the semi-automated Cumulus, and the fully-automated ImageJ-based approach) and three fully-automated volumetric methods (Volpara, Quantra and single energy x-ray absorptiometry (SXA)) were assessed in 3168 FFDM images from 414 cases and 685 controls. Linear regression models were used to assess associations between breast cancer risk factors and density among controls, and logistic regression models to assess density-breast cancer risk associations, adjusting for age, body mass index (BMI) and reproductive variables.

**Results:**

Quantra and the ImageJ-based approach failed to produce readings for 4% and 11% of the participants. All six density assessment methods showed that percent density (PD) was inversely associated with age, BMI, being parous and postmenopausal at mammography. PD was positively associated with breast cancer for all methods, but with the increase in risk per standard deviation increment in PD being highest for Volpara (1.83; 95% CI: 1.51 to 2.21) and Cumulus (1.58; 1.33 to 1.88) and lower for the ImageJ-based method (1.45; 1.21 to 1.74), Quantra (1.40; 1.19 to 1.66) and SXA (1.37; 1.16 to 1.63). Women in the top PD quintile (or BI-RADS 4) had 8.26 (4.28 to 15.96), 3.94 (2.26 to 6.86), 3.38 (2.00 to 5.72), 2.99 (1.76 to 5.09), 2.55 (1.46 to 4.43) and 2.96 (0.50 to 17.5) times the risk of those in the bottom one (or BI-RADS 1), respectively, for Volpara, Quantra, Cumulus, SXA, ImageJ-based method, and BI-RADS (*P* for trend <0.0001 for all). The ImageJ-based method had a slightly higher ability to discriminate between cases and controls (area under the curve (AUC) for PD = 0.68, *P* = 0.05), and Quantra slightly lower (AUC = 0.63; *P* = 0.06), than Cumulus (AUC = 0.65).

**Conclusions:**

Fully-automated methods are valid alternatives to the labour-intensive "gold standard" Cumulus for quantifying density in FFDM. The choice of a particular method will depend on the aims and setting but the same approach will be required for longitudinal density assessments.

**Electronic supplementary material:**

The online version of this article (doi:10.1186/s13058-014-0439-1) contains supplementary material, which is available to authorized users.

## Introduction

Mammographic density is one of the strongest breast cancer risk factors [1],[2], which is being increasingly used to tailor preventive and screening strategies to a woman's risk. It is also a major determinant of sensitivity of mammographic screening and, thus, of interval cancer rates [3],[4]. Consequently, in many US states, it is now mandatory to inform screening-attendees of their density.

There are several area-based [5]-[10] and volumetric [11]-[14] approaches to measuring density in screen-film mammography, but the quantitative semi-automated Cumulus approach is regarded as the gold standard, as its area-based measurements have consistently been shown to be strongly associated with breast cancer risk [2]. Screen-film mammography is gradually being replaced by full-field digital mammography (FFDM), and fully automated volumetric methods have been developed for density assessment on digital images [15]-[17] but to date, evaluation of their performance has been limited to establishing whether their measurements correlate with those from more established methods, such as BI-RADS or Cumulus, or to evaluating the extent to which they are associated with breast cancer risk factors [15]-[18].

We conducted the first comparison of the ability of six methods of mammographic density measurement to predict breast cancer risk, including well-established analogue methods adapted for, and novel methods developed for FFDM.

## Methods

### Study population

Cases were women with newly diagnosed breast cancer in the Royal Marsden Hospital (RMH), London, between April 2010 and July 2012. Controls were women who attended routine screening at the Central and East London Breast Screening Service (CELBSS) during the same period and were found to be breast cancer free. CELBSS is part of the England and Wales national mammographic screening programme offered once every three years to women aged 50 (47 from 2012) to 70 years (older women can self-refer) [19]. Women with a history of breast or ovarian cancer, or with breast implants, were excluded. The study was approved by all relevant ethics committees (Research Ethics Committees from the Royal Marsden Hospital, the Barts and the London NHS Trust, and the London School of Hygiene and Tropical Medicine). Participants provided written informed consent.

### Data collection

Data on breast cancer risk factors (Table 1) were collected by questionnaire at the time of screening for controls and after diagnostic confirmation for cases (up to 15½ months after mammography), and complemented with data from clinical records. Participants underwent two-view (standard cranio-caudal (CC) and medio-lateral oblique (MLO)) FFDM on each breast using Senographe DS units (GE Healthcare, Slough, England).

**Table 1:** **Baseline characteristics of the participants by case–control status**

	Controls (n = 685)		Cases (n = 414)	
**Age, years at mammography**				
Mean (SD)	59.5 (6.6)		67.5 (12.7)	
Missing, n	6		2	
**Ethnicity**				
White, n (%)	520 (76.5)		370 (90.5)	
Other, n (%)	160 (23.5)		39 (9.5)	
Missing, n	5		5	
**Educational level**				
None, n (%)	15 (2.2)		10 (3.7)	
Primary, n (%)	20 (3.0)		7 (2.6)	
Secondary, n (%)	297 (43.9)		186 (68.4)	
University, n (%)	344 (50.9)		69 (25.4)	
Missing, n	9		142	
**Body mass index at mammography** ^**a**^ **, kg/m** ^**2**^				
Mean (SD)	26.1 (5.6)		26.4 (4.9)	
Missing, n	29		46	
**Age at menarche, yrs**				
<12, n (%)	141 (21.0)		81 (21.3)	
12, n (%)	130 (19.4)		78 (20.5)	
13, n (%)	168 (25.0)		87 (22.8)	
14+, n (%)	232 (34.6)		135 (35.4)	
Missing, n	14		33	
**Menopausal status at mammography** ^**b**^				
Pre- and perimenopausal, n (%)	91 (13.3)		55 (13.3)	
Postmenopausal, n (%)	591 (86.7)		358 (86.7)	
Missing, n	3		1	
**Ever use of oral contraceptives**				
Yes, n (%)	472 (70.5)		216 (55.2)	
No, n (%)	198 (29.6)		175 (44.8)	
Missing, n	15		23	
**Ever use of hormonal therapy**				
Yes, n (%)	208 (31.2)		143 (36.8)	
No, n (%)	459 (68.8)		246 (63.2)	
Missing, n	18		25	
**Nulliparity**				
No, n (%)	467 (69.1)		343 (84.1)	
Yes, n (%)	209 (30.9)		65 (15.9)	
Missing, n	9		6	
**Number of children** ^**c**^ **, n (%)**				
1 to 2	301 (65.4)		218 (64.9)	
2 to 3	126 (27.4)		105 (31.3)	
5+	33 (7.2)		13 (3.9)	
**Age at first birth, yrs** ^**c**^ **, n (%)**				
<20	83 (18.1)		25 (7.7)	
20 to 30	258 (56.3)		213 (65.7)	
30+	117 (25.6)		86 (26.5)	
**Ever breastfed** ^**c**^ **, n (%)**				
Yes	358 (77.2)		224 (74.7)	
No	106 (22.8)		76 (25.3)	

^a^Body mass index estimated from self-reported height and weight as weight/height^2^ (in kg/m^2^). ^b^Postmenopausal women defined as those who self-reported natural (that is, cessation of menses for at least 12 months) or surgical menopause, were older than 55 years, or ever used hormone replacement therapy. Due to small numbers pre- (that is, younger than 55 years and still having regular periods) and perimenopausal (that is, younger than 55 years and having irregular periods) women were combined into a single category. ^**c**^Restricted to ever-parous women.

**Figure 1:**
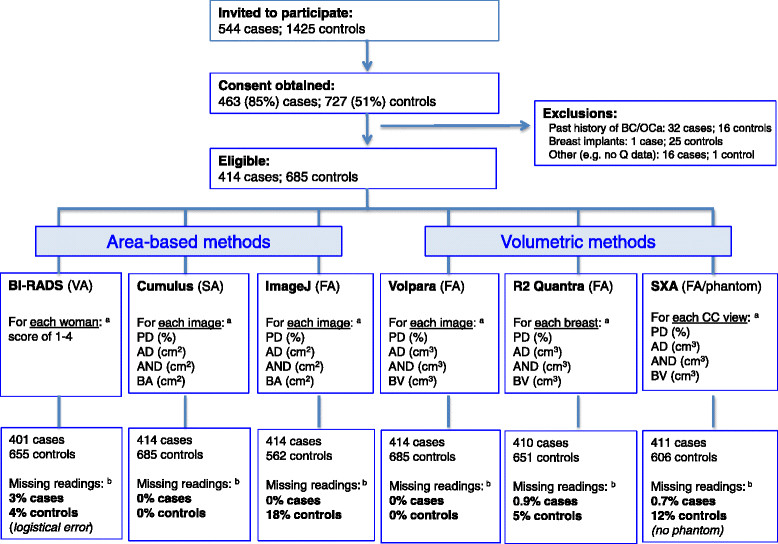
**Flowchart detailing the recruitment of study participants.**^a^Images from both breasts in controls, and from the unaffected contralateral breast only for cases. ^b^Percentage of women with missing readings. AD, absolute area or volume of dense tissue; AND, absolute area or volume of non-dense tissue; BS, breast size (area or volume); BC, breast cancer; FA, fully-automated method; OCa, ovarian cancer; PD, percent density; SA, semi-automated method; VA, visual assessment.

Density readings were performed on anonymised images from both breasts in controls and from the unaffected breast for cases (Figure 1). The area-based methods comprised: (i) visual assessment by two radiologists (SA, SV) who together examined all the unaffected processed images from each woman and gave a single BI-RADS score (1: percent density (PD) <25%; 2: PD = 25 to 50%; 3: PD = 51 to 75%; 4: PD >75%) [5]. A subset of 62 films was re-read independently by the same two readers with an interval of ≥6 months between the two readings; (ii) semi-automated interactive threshold Cumulus v3 [6],[7], after conversion of raw digital images into analogue-like ones. Readings were performed by a single observer (IdSS) in batches, each containing a 7% random sample of all participants as duplicates to allow assessment of intra-observer reliability; and (iii) the ImageJ-based method, a fully-automated approach, which attempts to mimic Cumulus [8],[20], after conversion of processed images into analogue-like ones. The latter two methods estimated area-based breast size, absolute density, absolute non-density (all in cm^2^) and PD, separately for each image. From raw images the volumetric methods, which were all fully-automated, estimated breast, absolute dense and absolute non-dense volumes (all in cm^3^), and PD. They comprised: (i) Volpara v1.0 (Matakina Technology Limited, Wellington, New Zealand) [18], which yielded separate density estimates for each CC/MLO image; (ii) R2 Quantra v1.3 (Hologic, Bedford, MA, USA) [17], which combined the information from both views to produce average estimates for each unaffected breast; and (iii) the single energy x-ray absorptiometry (SXA) method, v6.5 [12], which required the fitting of a calibration phantom onto the compression paddle of the x-ray machine and which to date, can only process CC images (see Additional file 1 for further details on the various density assessment methods).

### Statistical methods

Appropriate transformations (square root for area-based metrics and natural-log transformation for volumetric metrics) were applied to normalise the distributions generated by the five quantitative methods. Scatter and Bland-Altman plots were used to compare the transformed distributions separately for each view and breast combination. Intra-method reliability (intraclass correlation coefficient, ICC) of a single density value (left or right image of a CC or MLO view), and of the left-right average for that view, was estimated as the percentage of the total variance due to between-subject variance among control women. Intra-observer BI-RADS agreement was assessed using weighted *κ* statistic (weights of 1, 0.67, 0.33 and 0 for categories 1 to 4 apart). Inter-method correlation and rank agreement was assessed by estimating the Spearman's rank correlation coefficient (*r*) and the proportion of control women classified in the same, or the same ±1 adjacent, quintile.

Associations of breast cancer risk factors with PD, and absolute density and non-density, were assessed among controls, by linear regression models adjusting for age, body mass index (BMI) and reproductive variables. Regression coefficients represent the difference in each density measure (in number of SDs on the transformed scale) associated with a unit change in the explanatory variable.

Logistic regression models were fitted to examine associations between density and breast cancer risk, adjusting for age, BMI, and reproductive variables (further adjustment for ethnicity did not affect the results). For the quantitative methods, the density measurements from the unaffected breast for cases and a randomly selected breast for controls were included in the models as continuous variables (in SD scores) or as quintiles (defined among controls). Sensitivity analyses included estimates by view; restriction to participants with density readings available for all quantitative methods; restriction to those aged <80 years; and use of multiple imputation methods to impute values for women with missing confounder data. The area under the curve (AUC) of the receiver operating characteristic curve was used to compare the ability of the various quantitative methods to discriminate between cases and controls. Analyses were performed in Stata 13.1 [21]. All *P*-values are two-sided.

## Results

In all, 463 cases and 727 controls were recruited (response rate: 85% for cases, 51% for controls), but only 414 cases and 685 controls were eligible (Figure 1). Cases were older and more likely to be of white ethnicity than controls (Table 1). Volpara and Cumulus produced readings for all participants; missing readings for BI-RADS and SXA were caused by logistical errors whereas those for the ImageJ-based and Quantra approaches were intrinsic failures of these methods (Figure 1). Women with missing readings from the ImageJ-based, Quantra or SXA methods did not differ from those with such readings in terms of their age, BMI or reproductive factors but on average, those with missing ImageJ-based readings had lower Cumulus PD (median (inter-quartile range): 2.5% (0.8 to 5.6%) for women with missing versus 8.7% (2.8 to 23.6%) for those without missing readings, *P* <0.0001).

### Inter- and intra-method comparisons among controls

**Figure 2:**
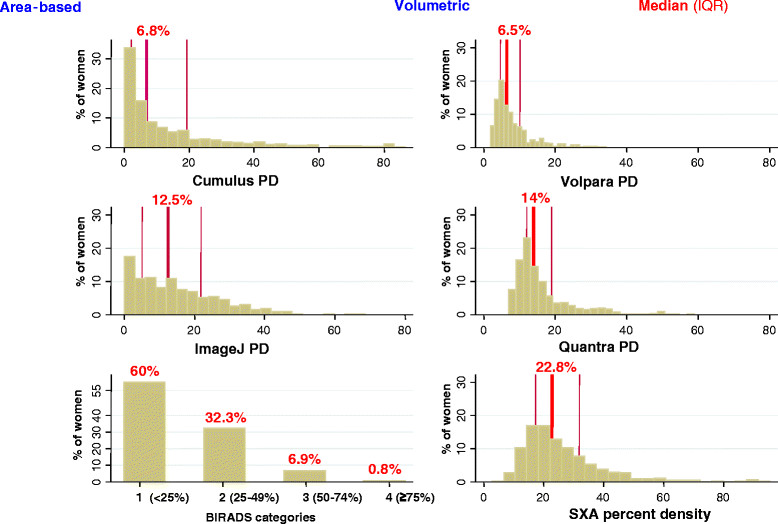
**Distribution of control participants by BI-RADS categories and percent density (PD) values yielded by each quantitative method.** *Density readings taken on the left cranio-caudal view (CC) except for BI-RADS, for which the four breasts/views were used to provide a single score per woman, and Quantra, which aggregated data from the CC and medio-lateral oblique view to provide a single measurement per breast.

PD distributions from the five quantitative methods were right-skewed, particularly for the area-based approaches which included a high proportion of women with zero values (no measurable dense tissue) (Figure 2). Relative to Cumulus, the ImageJ-based method yielded higher PD estimates (Figure 2) due to overestimation of absolute density and underestimation of breast area (see Additional file 2: Figures S1, S2). Volumetric methods yielded narrower PD distributions with no zero values. SXA produced the highest estimates and Volpara the lowest (Figure 2), paralleling similar between-method differences in the estimation of absolute density (see Additional file 2: Figures S1, S2). Over 92% of controls were classified as BI-RADS 1 to 2 (Figure 2).

PD measurements from the four fully-automated methods were strongly correlated with those produced by the semi-automated Cumulus (*r* >0.77 for all; see Additional file 2: Figure S3), mainly driven by strong correlations for breast size (*r* >0.93 for all; Additional file 2: Figure S5) as the corresponding correlations for absolute density were weaker (*r* ≤0.41 except, as expected, for strong ImageJ-based versus Cumulus correlation (*r* = 0.90)); Additional file 2: Figure S4). Pair comparisons of PD values yielded by the quantitative methods were high (*r* from 0.76 (SXA-Volpara) to 0.92 (ImageJ-Cumulus)) (see Additional file 3: Table S1), with 47% and 66% of the controls being classified in the same quintile and between 87% and 97% in the same ±1 quintile (see Additional file 3: Table S2).

The PD distributions across breasts and views had a similar shape but estimates were slightly higher for the right breast for all quantitative methods, and for the CC view for those that produced readings for both views (see Additional file 3: Table S3), reflecting mainly between breast/view differences in breast size. Both intra-observer agreement for BI-RADS (*k* >0.80) and intra-reader reliability for Cumulus (99% for breast area, 90% for PD and 87% for dense area) were high. The reliability of PD measurements based on a single film (ICC >0.84 for all) and on the left-right mean (ICC >0.91) were high for all quantitative methods regardless of view, driven by very high reliability for breast size (ICC for all methods: >0.93 for a single film; >0.96 for left-right mean) but somewhat lower for absolute density (see Additional file 3: Table S3).

### Associations with breast cancer risk factors among controls

**Figure 3:**
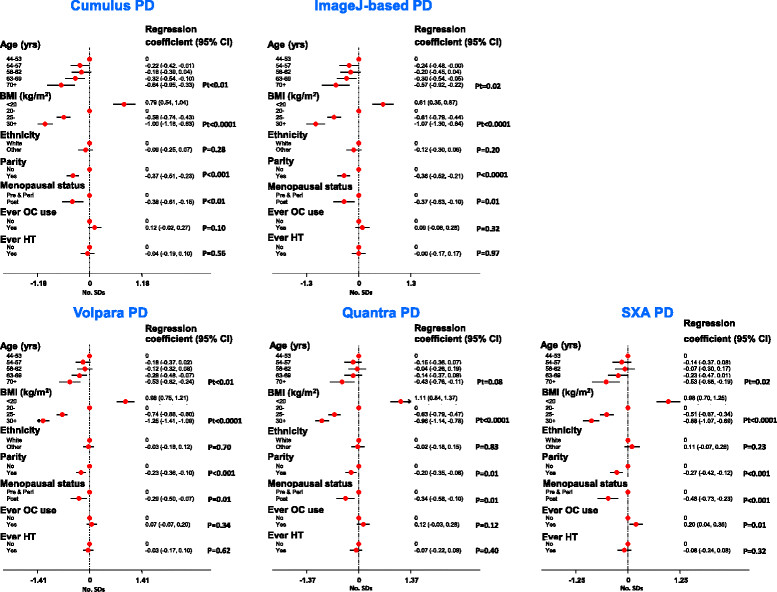
**Mutually-adjusted associations of known determinants of mammographic density with percent density (PD) readings in control women.** PD readings are the mean of four breast/view readings per woman (except for Quantra and single x-ray absorptiometry - see Methods). BMI, body mass index; HT, hormonal therapy; OC, oral contraceptives; Pt, *P* for linear trend.

The direction and magnitude (in SD numbers) of the PD associations with breast cancer risk factors were remarkably similar across the five quantitative methods, and in the direction expected, given the effects of these variables on risk (Figure 3). All five quantitative methods showed PD to be inversely associated with age. For Cumulus, this age trend in PD reflects both an age decrease in absolute density and an age increase in absolute non-density, whereas for Volpara and the ImageJ-based method there was only an age decrease in absolute density, and for SXA only an increase in absolute non-density (see Additional file 2: Figures S6, S7). For all methods there was a strong inverse association of PD with BMI, driven by positive association of BMI with absolute non-density for all methods, as well as negative association of BMI with absolute density for the two area-based methods. In contrast, a trend of increasing dense volume with increasing BMI was observed for all volumetric methods. PD was lower among parous women for all quantitative methods, reflecting reductions in absolute density and also, for the two area-based methods only, increases in absolute non-density. PD was also lower among postmenopausal women for all methods, driven by parallel declines in absolute density. Ever-use of oral contraceptives was positively associated with increases in absolute density, but only significantly so for the volumetric methods. There were no associations with ever-use of hormonal therapy (Figure 3; see Additional file 2: S6, S7), ages at menarche or first birth, or educational level (not shown).

Mutually adjusted analyses showed that the odds of having a BI-RADS score ≥3 relative to a score <3 decreased with increasing age (*P* for linear trend (Pt) = 0.06), increasing BMI (Pt <0.0001) and being parous versus nulliparous (odds ratio (OR): 0.38; 95% CI 0.20, 0.74).

### Breast cancer risk

**Figure 4:**
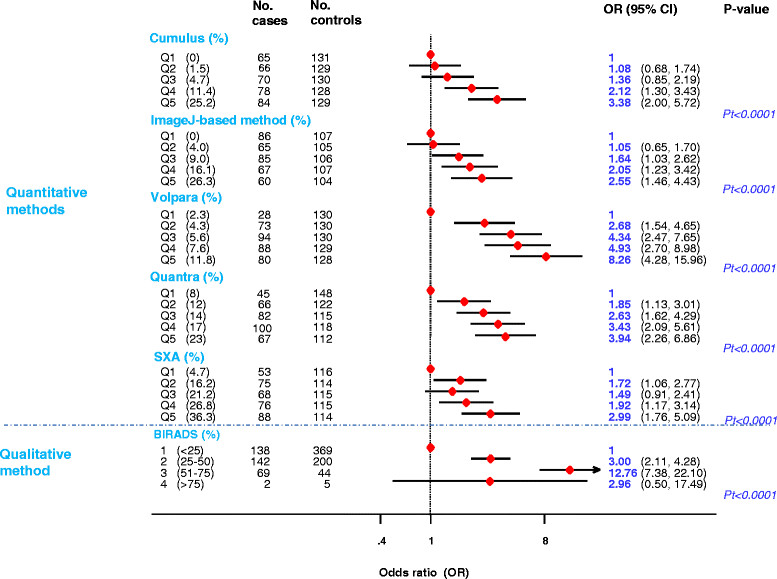
**Breast cancer risk by fifths of percent density for each quantitative method, and by BI-RADS categories.** Fifths of percent density risk were defined by quintiles of the density distributions among controls. Pt, *P* for linear trend; OR, odds ratio; SXA, single energy x-ray absorptiometry.

All methods produced positive associations between PD and breast cancer risk. Women in the top PD quintile had 3.38 (95% CI 2.00, 5.72), 2.55 (1.46, 4.43), 8.26 (4.28, 15.96), 3.94 (2.26, 6.86) and 2.99 (1.76, 5.09) times the risk of those in the bottom one, respectively, for Cumulus, ImageJ-based method, Volpara, Quantra and SXA (Pt <0.0001 for all; Figure 4). The SXA OR was based on CC, rather than CC-MLO average values but the equivalent Volpara OR for the CC view was 6.18 (3.3, 11.42). The gradient in risk across quintiles was steeper for Volpara, partly due to it being better at identifying women at low risk than the area-based methods as demonstrated by the lower number of cases that fell in the bottom quintile. There was also a strong positive trend in risk across BI-RADS categories but the magnitude of this cannot be compared with those from the quantitative methods (for example, BI-RADS score 1 encompassed the bottom four Cumulus quintiles).

**Figure 5:**
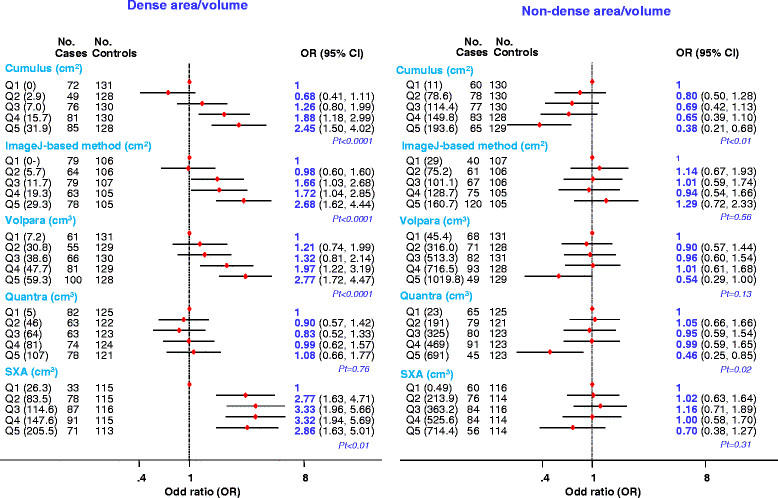
**Breast cancer risk by fifths of absolute density and non-density for each quantitative method as defined by quintiles of the distributions in controls.** OR, odds ratio; Pt, *P* for linear trend; SXA, single energy x-ray absorptiometry.

The positive association of risk with Cumulus-measured PD reflected a positive association of risk with absolute density and, to a lesser extent, a negative association of risk with absolute non-density (Figure 5). In contrast, for the ImageJ-based method, Volpara and SXA the positive associations of risk with PD reflected mainly positive associations of risk with absolute density, whereas for Quantra it reflected mainly a negative association of risk with absolute non-density (Figure 5).

**Figure 6:**
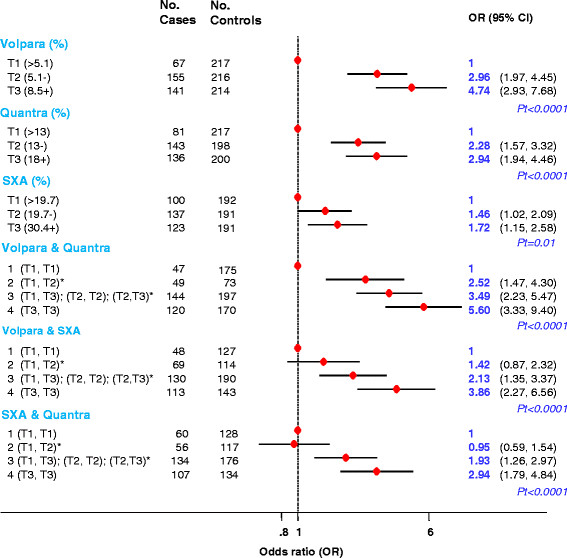
**Breast cancer risk associated with aggregated scores produced by combining readings from two fully-automated volumetric methods.** *Aggregated categories based on tertiles as defined among control women: 1: if classified in the bottom tertile (T1) by both methods; 2: if classified in T1 by one method but in the middle tertile (T2) by the other; 3: if classified in T1 by one method but in the top tertile (T3) by the other, or in T2 by both methods, or in T2 by one method but in T3 by the other; 4: if classified in T3 by both methods. OR, odds ratio; Pt, *P* for linear trend; SXA, single energy x-ray absorptiometry.

Combining readings from pairs of fully-automated volumetric methods did not affect the magnitude of the PD-risk associations (Figure 6).

Risk increases per one SD increase in average PD were consistently higher for Cumulus (1.58; 95% CI 1.33, 1.88) than the ImageJ-based method (1.45; 1.21, 1.74), and for Volpara (1.83; 1.51, 2.21) than the other volumetric methods, Quantra (1.40; 1.19, 1.66) and SXA (1.37; 1.16, 1.63) (Table 2). The increases in risk associated with one SD increase in absolute density were as high as those associated with an equivalent increase in PD for the area-based methods, but lower for the volumetric methods. These findings were robust to a range of sensitivity analyses (Table 2).

**Table 2:** **Mammographic density and breast cancer risk**

	Area-based methods						Volumetric-based methods								
	Cumulus			ImageJ-based method			Volpara			Quantra			SXA		
Percent density	Number	OR^a^	(95% CI)	Number	OR^a^	(95% CI)	Number	OR^a^	(95% CI)	Number	OR^a^	(95% CI)	Number	OR^a^	(95% CI)
Average of CC and MLO readings^b^	1,010	**1.58**	(1.33, 1.88)	891	**1.45**	(1.21, 1.74)	1,010	**1.83**	(1.51, 2.21)	-	-	-	-	-	**-**
Only CC readings^b^	1,009	**1.57**	(1.32, 1.87)	888	**1.33**	(1.11, 1.59	1,009	**1.75**	(1.45, 2.10)	975	**1.40**	(1.19, 1.66)	934	**1.37**	**(1.16, 1.63)**
Only MLO readings^b^	1,008	**1.56**	(1.31, 1.85)	882	**1.42**	(1.19, 1.69)	1,008	**1.75**	(1.44, 2.11)				-	-	**-**
Multiple imputation^c^	1,095	**1.63**	(1.37, 1.93)	971	**1.45**	(1.22, 1.73)	1,095	**1.82**	(1.51, 2.20)	1,057	**1.40**	(1.19, 1.64)	1,014	**1.35**	**(1.15, 1.60)**
Restricted to women aged <80 yrs	948	**1.58**	(1.32, 1.88)	830	**1.44**	(1.20, 1.72)	948	**1.78**	(1.47, 2.15)	914	**1.38**	(1.17, 1.63)	874	**1.36**	**(1.15, 1.62)**
Restricted to women with data for all quantitative methods	797	**1.55**	(1,29, 1.87)	797	**1.52**	(1.26, 1.84)	797	**1.64**	(1.33, 2.02)	797	**1.26**	(1.05, 1.55)	797	**1.48**	**(1.22, 1.80)**
**Dense area/volume**	**Number**	**OR** ^**a**^	**(95% CI)**	**Number**	**OR** ^**a**^	**(95% CI)**	**Number**	**OR**	**(95% CI)**	**Number**	**OR**	**(95% CI)**	**Number**	**OR**	**(95% CI)**
Average of CC and MLO readings^b^	1,010	**1.57**	(1.33, 1.84)	891	**1.56**	(1.31, 1.84)	1,010	**1.46**	(1.25, 1.70)	-	-	-	-	-	-
Only CC readings^b^	1,009	**1.58**	(1.35, 1.86)	888	**1.45**	(1.23, 1.71)	1,009	**1.47**	(1.26, 1.72)	975	**1.04**	(0.88, 1.22)	934	**1.26**	(1.08, 1.47)
Only MLO readings^b^	1,008	**1.52**	(1.30, 1.79)	882	**1.49**	(1.26, 1.77)	1,008	**1.38**	(1.18, 1.61)				-	-	-
Multiple imputation^c^	1,095	**1.60**	(1.36, 1.87)	971	**1.54**	(1.31, 1.82)	1,095	**1.43**	(1.23, 1.66)	1,057	**1.02**	(0.87, 1.19)	1,014	**1.23**	(1.05, 1.43)
Restricted to women aged <80 yrs	948	**1.57**	(1.33, 1.84)	830	**1.54**	(1.31, 1.82)	948	**1.49**	(1.27, 1.74)	914	**1.06**	(0.89, 1.26)	874	**1.27**	(1.08, 1.49)
Restricted to women with data for all quantitative methods	797	**1.63**	(1.36, 1.95)	797	**1.62**	(1.36, 1.94)	797	**1.70**	(1.42, 2.04)	797	**1.23**	(1.02, 1.49)	797	**1.74**	(1.43, 2.11)
**Non-dense area/volume**	**Number**	**OR**	**(95% CI)**	**Number**	**OR**	**(95% CI)**	**Number**	**OR**	**(95% CI)**	**Number**	**OR**	**(95% CI)**	**Number**	**OR**	**(95% CI)**
Average of CC and MLO readings^b^	1,010	**0.69**	(0.57, 0.84)	891	**1.13**	(0.93, 1.37)	1,010	**0.85**	(0.71, 1.03)	-	-	-	-	-	-
Only CC readings^b^	1,009	**0.74**	(0.61, 0.90)	888	**1.15**	(0.95, 1.40)	1,009	**0.88**	(0.73, 1.06)	975	**0.80**	(0.66, 0.97)	934	**0.90**	(0.75, 1.08)
Only MLO readings^b^	1,008	**0.66**	(0.54, 0.80)	882	**1.08**	(0.89, 1.30)	1,008	**0.84**	(0.69, 1.01)				-	-	-
Multiple imputation^c^	1,095	**0.67**	(0.56, 0.82)	971	**1.09**	(0.90, 1.31)	1,095	**0.83**	(0.69, 1.01)	1,057	**0.78**	(0.65, 0.94)	1,014	**0.87**	(0.73, 1.04)
Restricted to women aged <80 yrs	948	**0.69**	(0.57, 0.84)	831	**1.13**	(0.93, 1.37)	948	**0.88**	(0.72, 1.07)	914	**0.82**	(0.68, 1.00)	874	**0.91**	(0.76, 1.10)
Restricted to women with data for all quantitative methods	797	**0.93**	(0.74, 1.17)	797	**1.12**	(0.91, 1.37)	797	**1.10**	(0.88, 1.38)	797	**1.03**	(0.83, 1.28)	797	**1.01**	(0.81, 1.26)

This table presents the change in breast cancer risk associated with one standard deviation (SD) increase in percent density, absolute density and absolute non-density associated with each one of the five quantitative methods. ^a^Odds ratios (OR) and 95% CI as estimated by logistic regression models based on standardized values of transformed (log for volume-based methods and square root for area based methods adjusted for age, body mass index (BMI), menopausal status and parity (see Methods). There was no evidence of departure from linearity. ^b^Based on density measurements taken from the unaffected breast for cases and a random breast side (left or right) for controls. Measurements for each of the two views were yielded by Cumulus, the ImageJ-based method and Volpara. Single energy x-ray absorptiometry (SXA) generated measurements only for the CC view. Quantra combined data from both views to produce a single overall measurement for each breast (Figure 1). ^c^Multiple imputation methods used to impute values for women with missing data on age, BMI, menopausal status and/or parity (see Table 1 and Methods). CC, cranio-caudal view; MLO, medio-lateral oblique view; OR, odds ratios (with 95% confidence intervals).

The ImageJ-based method had slightly better ability to discriminate between cases and controls of screening age (50 to 69 years) (AUC for PD, age, BMI and reproductive factors = 0.65, *P* = 0.05), and Quantra slightly poorer (AUC for PD = 0.63, *P* = 0.06), than Cumulus (AUC = 0.64) (see Additional file 3: Table S4). Similar AUC values were observed for absolute density (see Additional file 3: Table S4).

## Discussion

### Main findings

This study provides the most comprehensive comparison to date of the performance of alternative methods of measuring mammographic density in FFDM images, comprising both well-established and novel methods, neither of which had been validated as predictors of risk in these images. Despite differences in their density distributions, they all produced positive associations with risk, which were strongest for Volpara and Cumulus. These two methods were also the only ones to produce readings for all images. Failure to produce readings affects the power/precision of a study but, more worrying, women with missing ImageJ-based values had lower Cumulus PD than those for whom readings were available, a finding previously reported for analogue images [20]. Such differences in missing values would bias the estimation of the magnitude of the association between the ImageJ-based PD values and risk, as women with low PD values are more likely to be controls and therefore their exclusion leads to underestimation of the magnitude of the association. SXA readings were also missing for a substantial proportion of participants due to lack of a phantom, highlighting a practical limitation of this method when implemented in busy clinical settings (and the impossibility of applying it retrospectively to historical images).

The majority of the participants had, in line with their age and postmenopausal status, low PD and absolute density according to all methods. However, the distributions of the volumetric PD estimates were narrower than those of the area-based PD values, with the latter having a high percentage of zero values, consistent with findings in analogue images [20]. Area-based methods use an intensity threshold to simply dichotomize breast pixels as being completely (100%) dense or non-dense (0% dense), whereas volumetric methods quantify a continuous amount of dense tissue at each pixel. The ImageJ-based method aims to mimic the Cumulus approach [8], but it produced different density distributions and, consistently with previous observations in analogue images [20], its reliability was lower and the association with risk weaker. There were also differences in the density distributions produced by the three volumetric methods, with Quantra and SXA producing higher estimates. The lack of perfect between-method rank correlation/agreement, although not unexpected as these methods may capture different density dimensions, highlights the need to use the same approach in longitudinal density assessments.

Consistent with other studies [1],[22],[23], PD was lower in women who were older, parous, postmenopausal, or had higher BMI, with the magnitude of the effects (in SDs) being similar for all quantitative methods. The PD decline with increasing BMI reflected a strong positive trend of BMI with the non-dense area/volume of the breast, which was consistent across all quantitative methods. However, and akin to findings in analogue films [20],[23], whereas dense area was smaller at higher BMI, dense volume was larger. Dense volume is equivalent to volumetric PD multiplied by the total breast volume. Thus, although women with higher BMI have lower volumetric PD they also have larger breast volumes and, hence, higher dense volumes than those with lower BMI. All methods showed positive associations of PD with risk, but with the magnitude being greatest for Volpara and Cumulus. For Cumulus, the ImageJ-based method, Volpara, and SXA the positive risk association with PD reflected similar positive trends in risk with absolute density whereas for Quantra it reflected mainly a negative trend in risk with absolute non-density. The Quantra findings are difficult to explain as breast tumors arise predominantly within radio-dense tissue of the breast [24]. The ability to discriminate between cases and controls was low (AUC: approximately 63 to 69%) for all methods, albeit similar to that reported by others [8],[20],[25],[26], highlighting its limited value in individual risk prediction. However, mammographic density might be useful, alone or jointly with other risk factors, to stratify women in the population according to risk for tailored interventions (for example, screening).

The study did not aim to provide direct information on the ease of incorporating any of the four fully automated density methods into screening/clinical practice as it was designed to interfere as little as possible with the usual routine. However, a few logistic issues emerged. First, the methods were based on raw images, and thus, required routinely saving them, and having the electronic data storage capacity to do so. Currently, only processed images are routinely saved in most screening/clinical settings but ongoing efforts to develop fully automatic density measurement approaches for processed images may overcome this limitation in the future. Second, although none of the methods required the use of special equipment during image acquisition, with the exception of SXA as discussed above, their software requirements and output varied. Quantra (version 1.3) produced for each participant, at the time of mammography, a digital image with the density measurements super-imposed on it, which is convenient in screening/clinical settings, but not efficient in large-scale studies as the density measurements for analysis will have to be extracted manually from these images (Additional file 1). Different versions of Volpara are available - a clinical version, which provides readings during the examination, and a research version which is appropriate for large-scale collections. There are currently no stand-alone software packages for either the SXA or the ImageJ-based method, thus, limiting their widespread implementation.

### Strengths and limitations of the study

Strengths of this study include the large number of density assessment methods examined, the collection of covariates at the time of mammography, and the BI-RADS and Cumulus blind assessments. The study population was predominantly postmenopausal, thus, limiting the generalisability of the findings to premenopausal women. Similar between-method comparisons in younger women with denser breasts - for whom accurate risk stratification is more important - should be conducted. Response rates were low for healthy controls and information on breast cancer risk factors, including BMI, was self-reported; however, any potential bias is likely to have been non-differential as density is not routinely ascertained in clinical/screening settings in the UK, and would have affected all methods similarly. Analyses were based on diagnostic images from the unaffected breast for cases, an approach used by others [2]. Although masking may have led to underestimation of the true magnitude of the density-risk association [2], this would have affected all methods similarly. The volumetric methods examined here attempted to estimate volumetric density from two-dimensional images, supplemented by information on the third dimension (using phantoms, breast thickness, plate tilting). True three-dimensional x-ray breast imaging techniques, such as tomosynthesis or magnetic resonance imaging, are not widely used in clinical settings.

## Conclusions

Mammographic density offers the potential, alone or in combination with other genetic and non-genetic factors, to improve breast cancer risk prediction [25]; to target primary prevention efforts (for example, chemoprevention, lifestyle behavioural changes) [27],[28]; to tailor screening according to risk by identifying those who may benefit from more intensive screening and those for whom screening may be more harmful than beneficial [29]; and to monitor response to treatment and risk of adverse outcomes [30]. However, its applicability in clinical and screening settings has been hampered by the subjective and labour-intensive nature of BI-RADS [31]-[33] and Cumulus, the most widely-used density estimation methods. Cumulus has been shown to have high between- and within-reader reliability in research settings [23], in which efforts are made to train the readers and ensure standardisation of procedures, but it is unlikely that similar high inter-reader reliability values will be observed when Cumulus is used in clinical/screening practice. This study demonstrates that fully automated methods are valid alternatives for FFDM. The choice of a particular method will depend on the aims (for example, aetiological investigations versus risk prediction) and setting (for example, research versus clinical), but the same approach will be required in longitudinal assessments of density.

## Authors' information

The two first authors (Amanda Eng and Zoe Gallant) contributed equally. The three senior authors (Sarah Vinnicombe, Steve Allen and Isabel dos-Santos-Silva) contributed equally.

## Additional files

## Electronic supplementary material

Additional file 1: Description of the six mammographic density assessment methods.(DOCX 47 KB)

Additional file 2: Figure S1.: Distributions of absolute density estimates taken from the left cranio-cauldal (CC)* view for control women, by method (*except for Quantra, which aggregates data from the two views to provide a single measurement per breast). **Figure S2.** Distributions of breast area/volume estimates taken from the left CC* view for control women, by method (*except for Quantra, which aggregates data from the two views to provide a single measurement per breast). **Figure S3.** PD readings* from BI-RADS, ImageJ-based method, Volpara, Quantra and single energy x-ray absorptiometry (SXA) versus those from Cumulus in control women. *Mean of four breast/view readings per woman (except for Quantra and SXA - see Methods). Values are plotted on the appropriate transformed scale (see Methods). **Figure S4.** Absolute density readings* from BI-RADS, ImageJ-based method, Volpara, Quantra and SXA versus those from Cumulus in control women. *Mean of four breast/view readings per woman (except for Quantra and SXA - see Methods). Values are plotted on the appropriate transformed scale (see Methods). **Figure S5.** Breast area/volume readings* from BI-RADS, ImageJ-based method, Volpara, Quantra and SXA versus those from Cumulus. *Mean of four breast/view readings per woman (except for Quantra and SXA - see Methods). Values are plotted on the appropriate transformed scale (see Methods). **Figure S6.** Mutually adjusted association of breast cancer risk factors with absolute density readings* in control women, by method. HT, hormonal therapy; OC, oral contraceptives; Pt, *P* for linear trend. *Mean of four breast/view readings per woman (except for Quantra and SXA - see Methods). **Figure S7.** Mutually adjusted association of breast cancer risk factors with absolute non-density readings* in control women, by method. HT, hormonal therapy; OC, oral contraceptives; Pt, *P* for linear trend. *Mean of four breast/view readings per woman (except for Quantra and SXA - see Methods). (PPTX 399 KB)

Additional file 3: Table S1.: Spearman's rank correlation coefficients (*r*) between the various quantitative methods of quantifying percent density in control women. **Table S2.** Quintile agreement between the five quantitative methods of quantifying percent density in control women. **Table S3.** Distribution of density readings and level of agreement by method, breast and view in control women. **Table S4.** Area under the receiving operating curve (AUC) for percent and absolute density for each quantitative method. (DOCX 47 KB)

Below are the links to the authors’ original submitted files for images.

Authors’ original file for figure 1

Authors’ original file for figure 2

Authors’ original file for figure 3

Authors’ original file for figure 4

Authors’ original file for figure 5

Authors’ original file for figure 6

## Abbreviations

AUC: area under the curve
BMI: body mass index
CC: cranio-caudal view
FFDM: full-field digital mammography
HT: hormone therapy
ICC: intra-class correlation coefficient
MLO: medio-lateral oblique view
OC: oral contraceptives
OR: odds ratio
PD: percent density
Pt: *P* for linear trend
SXA: single energy x-ray absorptiometry
