# Supplementary material for: Digital mammographic density and breast cancer risk: a case–control study of six alternative density assessment methods
Source: Breast Cancer Res. 2014 Sep 20;16:439. doi: 10.1186/s13058-014-0439-1 (PMC4303120; doi:10.1186/s13058-014-0439-1)
Supplement: Supplementary file 1 — Additional file 1: Description of the six mammographic density assessment methods.(DOCX 47 KB) [file 13058_2014_439_MOESM1_ESM.docx]

**Additional Methods: Description of the six mammographic density assessment methods**

**BI-RADS** is a qualitative visual assessment of area-based density which classifies women into four categories based on the subjective analysis of patterns observed on the mammogram: 1: almost entirely fatty, glandular (dense) tissue <25% of the total breast area; 2: scattered fibro-glandular densities, density between 25%-50%; 3: heterogeneously dense, density between 51%-75%; and 4: extremely dense, density >75%) [[1](#_ENREF_1)]. The readings were performed by two radiologists (SA, SV) in batches of anonymised unaffected images for ~150 participants. A subset of 62 films was re-read independently with a ≥6 month-interval between the two readings.

**Cumulus** (Cumulus 4 software, University of Toronto) is a semi-automated quantitative density estimation method originally-developed for digitised screen-film (analogue) images [[2](#_ENREF_2), [3](#_ENREF_3)]. As this method was originally developed for analogue images, the raw digital images from the present study were converted into “analogue-like” ones. The "For Processing" (raw) digital mammograms were converted to appearing to be digitized film using EasyScanConverter version 1.0 (Highnam Associates Limited). The software firstly simulated a film-like exposure, by simulating an automatic exposure control, and then simulated a digitization of that film using a high-end digitizer, notably a Lumisys 85. The “analogue-like” images were then displayed on a computer screen and the observer first delimited the breast from the background and then selected an intensity threshold to distinguish dense from non-dense pixels. Cumulus used the selected thresholds to automatically calculate the number of pixels in each area and to estimate percent density (PD) separately for each cranio-caudal (CC) and medio-lateral oblique (MLO) image. Breast size and absolute density, in cm^2^, were estimated by using an appropriate conversion factor. Absolute non-density, in cm^2^, was calculated as the difference between breast size and absolute density. The readings were performed by one of single observer (IdSS) in batches of anonymised images for about ~150 participants with each batch including images from a similar proportion of cases and controls. Each batch also contained a 7% random sample of all participants as duplicates to allow assessment of intra-observer reliability.

The **ImageJ-based method,** which is based on a public domain Java image processing program, ImageJ version 1.45s, is a recently developed fully-automated method which attempts to mimic Cumulus [[4](#_ENREF_4), [5](#_ENREF_5)]. Briefly, this method measures several image parameters and chooses those shown to predict Cumulus density in a training set of images of a given size with known Cumulus-density readings. The selected parameters are then included in a regression model to distinguish dense from non-dense areas of the breast, and estimate percent density values. Similarly to Cumulus, the ImageJ-based method was developed for analogue images and therefore the processed digital images in this study were first converted into “analogue-like” images using an ImageJ method specifically developed for this purpose. Objects on the image were defined as particles; those below a prior‑assigned pixel threshold value were removed (2500 pixels), and enhance contrast was applied to convert a raw image into an analogue‑like one. Training of mammographic density measurements was based on a random selection of 226 CC and 204 MLO images with known Cumulus readings. Similarly to Cumulus, the ImageJ-based method produces area-based estimates of breast size, absolute density and absolute non-density (all in cm^2^) as well as PD separately for each CC and MLO image. A copy of this software was made available to us (by JL). The readings for all participants were performed in one single batch at the end of the study.

**Volpara** (Volpara GUI v1.0, Algorithm 1.4, Matakina Technology Limited, Wellington, New Zealand) is a fully automated volumetric method which was developed as an extension of the method described by van Engeland et al. [[6](#_ENREF_6)]. The algorithm uses a physics model based on the x-ray image formation process to compute at each pixel in the raw digital image a measure of the x-ray attenuation and thereby the types and thicknesses of breast tissue in the cone of tissue between the pixel and the x-ray source. This method produces automated estimates of the volume of the breast and the volume of the dense fibroglandular tissue (both in cm^3^), as well as estimates of volumetric PD, for each CC and MLO image. The breast skin is not included in these estimations. A copy of this software was made available to us by Matakina Technology. The readings for all participants were performed in one single batch at the end of the study.

**R2 Quantra** (Hologic, Bedford, US), version 1.3, is a fully automated volumetric method which has evolved from the SMF method for analogue images [[7](#_ENREF_7), [8](#_ENREF_8)]. This algorithm is based on a physics model of the x-ray imaging process that estimates the amount of fibroglandular tissue that an x-ray must have penetrated at each pixel on the image in order to deposit the amount of energy measured by the detector. It then aggregates single pixel values to estimate the volume of the breast and the volume of the fibroglandular tissue. In contrast to Volpara, it incorporates the skin into its volume estimations and it combines the information from both the CC and the MLO views to produce average estimates of the breast volume, dense volume (both in cm^3^) and volumetric PD separately for the left and right breasts. Quantra was installed in each clinical setting. It produced for each participant, at the time of her mammography, a digital image with her density measurements super-imposed on it. These images were saved into a dedicated server and the density data subsequently extracted into a spreadsheet by two independent researchers, who were kept blind to the characteristics of the participants, and their datasets compared and checked.

**Single x-ray absorptiometry (SXA)** (University of California, San Francisco), version 6.5, is the third fully automated volumetric method to measure breast tissue composition from raw images. SXA compares the breast attenuation values against an in-image calibration phantom made from materials that mimic glandular-adipose tissue ratios [[9-11](#_ENREF_9)]. The phantom is mounted on the unused corner of the mammographic compression paddle. An algorithm then analyses the digital image and estimates breast height and amount of fibroglandular density at each pixel. The pixel-specific estimates are then summed up to produce total breast estimates for dense tissue volume (both in cm^3^), as well as of volumetric PD. To date, only the algorithm for processing CC images is in use. The raw anonymised images were transferred in batches to the University of California where the automated SXA readings were performed (by JS and colleagues) without knowledge of the case-control status, or of any other characteristic, of the participants.

**References**

1. American College of Radiology: **BI-RADS Mammography Breast Imaging Atlas**. Reston, Va: American College of Radiology; 2003.

2. Byng JW, Boyd NF, Fishell E, Jong RA, Yaffe MJ: **The quantitative analysis of mammographic densities**. *Phys Med Biol* 1994, **39**:1629-1638.

3. Boyd NF, Byng JW, Jong RA, Fishell EK, Little LE, Miller AB, Lockwood GA, Tritchler DL, Yaffe MJ: **Quantitative classification of mammographic densities and breast cancer risk: results from the Canadian National Breast Screening Study**. *J Natl Cancer Inst* 1995, **87**:670-675.

4. Li J, Szekely L, Eriksson L, Heddson B, Sundbom A, Czene K, Hall P, Humphreys K: **High-throughput mammographic-density measurement: a tool for risk prediction of breast cancer**. *Breast Cancer Res* 2012, **14**:R114.

5. Sovio U, Li J, Aitken Z, Humphreys K, Czene K, Moss S, Hall P, McCormack V, dos-Santos-Silva I: **Comparison of fully and semi-automated area-based methods for measuring mammographic density and predicting breast cancer risk**. *Br J Cancer* 2014;**110**:1908-1916.

6. van Engeland S, Snoeren PR, Huisman H, Boetes C, Karssemeijer N: **Volumetric breast density estimation from full-field digital mammograms**. *IEEE Trans Med Imaging* 2006, **25**:273-282.

7. **McCormack VA**, Highnam R, Perry N, dos Santos Silva I: **Comparison of a new and existing method of mammographic density measurement: intramethod reliability and associations with known risk factors**. *Cancer Epidemiol Biomarkers Prev* 2007, **16**:1148-1154.

8. Aitken Z, McCormack VA, Highnam RP, Martin L, Gunasekara A, Melnichouk O, Mawdsley G, Peressotti C, Yaffe M, Boyd NF, dos Santos Silva I: **Screen-film mammographic density and breast cancer risk: a comparison of the volumetric standard mammogram form and the interactive threshold measurement methods**. *Cancer Epidemiol Biomarkers Prev* 2010, **19**:418-428.

9. Shepherd JA, Kerlikowske KM, Smith-Bindman R, Genant HK, Cummings SR: **Measurement of breast density with dual X-ray absorptiometry: feasibility**. *Radiology* 2002, **223**:554-557.

10. Shepherd JA, Herve L, Landau J, Fan B, Kerlikowske K, Cummings SR: **Novel use of single X-ray absorptiometry for measuring breast density**. *Technol Cancer Res Treat* 2005, **4**:173-182.

11. Malkov S, Wang J, Kerlikowske K, Cummings SR, Shepherd JA: **Single x-ray absorptiometry method for the quantitative mammographic measure of fibroglandular tissue volume**. *Med Phys* 2009, **36**:5525-5536.
