# Supplementary material for: Digital mammographic density and breast cancer risk: a case–control study of six alternative density assessment methods
Source: Breast Cancer Res. 2014 Sep 20;16:439. doi: 10.1186/s13058-014-0439-1 (PMC4303120; doi:10.1186/s13058-014-0439-1)
Supplement: Supplementary file 2 — Additional file 2: Figure S1.: Distributions of absolute density estimates taken from the left cranio-cauldal (CC)* view for control women, by method (*except for Quantra, which aggregates data from the two views to provide a single measurement per breast). Figure S2. Distributions of breast area/volume estimates taken from the left CC* view for control women, by method (*except for Quantra, which aggregates data from the two views to provide a single measurement per breast). Figure S3. PD readings* from BI-RADS, ImageJ-based method, Volpara, Quantra and single energy x-ray absorptiometry (SXA) versus those from Cumulus in control women. *Mean of four breast/view readings per woman (except for Quantra and SXA - see Methods). Values are plotted on the appropriate transformed scale (see Methods). Figure S4. Absolute density readings* from BI-RADS, ImageJ-based method, Volpara, Quantra and SXA versus those from Cumulus in control women. *Mean of four breast/view readings per woman (except for Quantra and SXA - see Methods). Values are plotted on the appropriate transformed scale (see Methods). Figure S5. Breast area/volume readings* from BI-RADS, ImageJ-based method, Volpara, Quantra and SXA versus those from Cumulus. *Mean of four breast/view readings per woman (except for Quantra and SXA - see Methods). Values are plotted on the appropriate transformed scale (see Methods). Figure S6. Mutually adjusted association of breast cancer risk factors with absolute density readings* in control women, by method. HT, hormonal therapy; OC, oral contraceptives; Pt, P for linear trend. *Mean of four breast/view readings per woman (except for Quantra and SXA - see Methods). Figure S7. Mutually adjusted association of breast cancer risk factors with absolute non-density readings* in control women, by method. HT, hormonal therapy; OC, oral contraceptives; Pt, P for linear trend. *Mean of four breast/view readings per woman (except for Quantra and SXA - see Methods). (PPTX 399 KB) [file 13058_2014_439_MOESM2_ESM.pptx]

## Slide 1
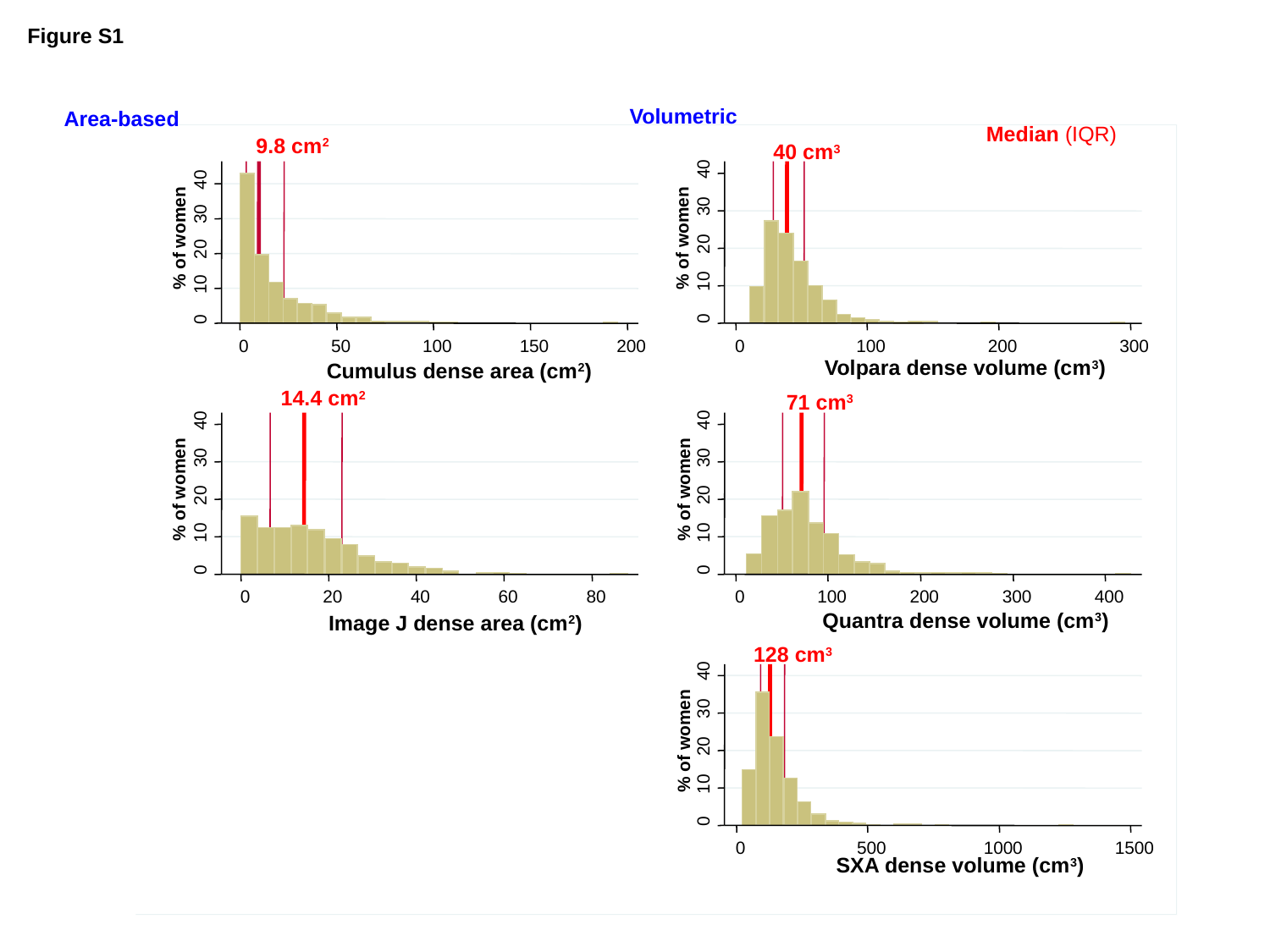

Figure S1
Volumetric
Area-based
Median (IQR)
40
40
30
30
% of women
% of women
20
20
10
10
0
0
0
50
100
150
200
0
100
200
300
Volpara dense volume (cm3)
Cumulus dense area (cm2)
40
40
30
30
% of women
% of women
20
20
10
10
0
0
0
20
40
60
80
0
100
200
300
400
Quantra dense volume (cm3)
Image J dense area (cm2)
40
30
% of women
20
10
0
0
500
1000
1500
SXA dense volume (cm3)
9.8 cm2
 40 cm3
 14.4 cm2
 71 cm3
 128 cm3

## Slide 2
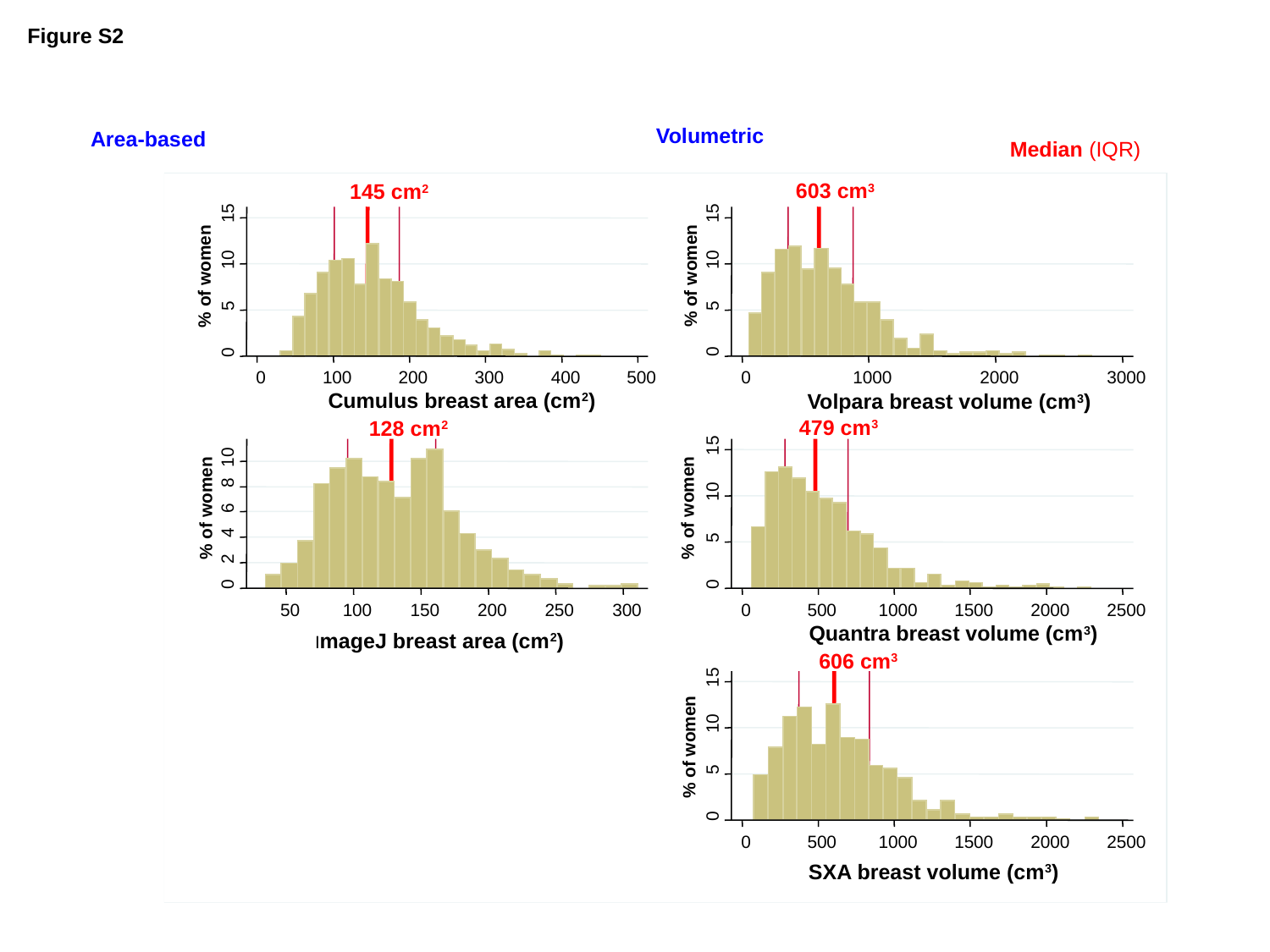

Figure S2
Volumetric
Area-based
Median (IQR)
15
15
10
10
% of women
% of women
5
5
0
0
0
100
200
300
400
500
0
1000
2000
3000
Cumulus breast area (cm2)
Volpara breast volume (cm3)
10
8
6
% of women
4
2
0
50
100
150
200
250
300
ImageJ breast area (cm2)
15
10
% of women
5
0
0
500
1000
1500
2000
2500
Quantra breast volume (cm3)
15
10
% of women
5
0
0
500
1000
1500
2000
2500
SXA breast volume (cm3)
603 cm3
145 cm2
479 cm3
128 cm2
606 cm3

## Slide 3
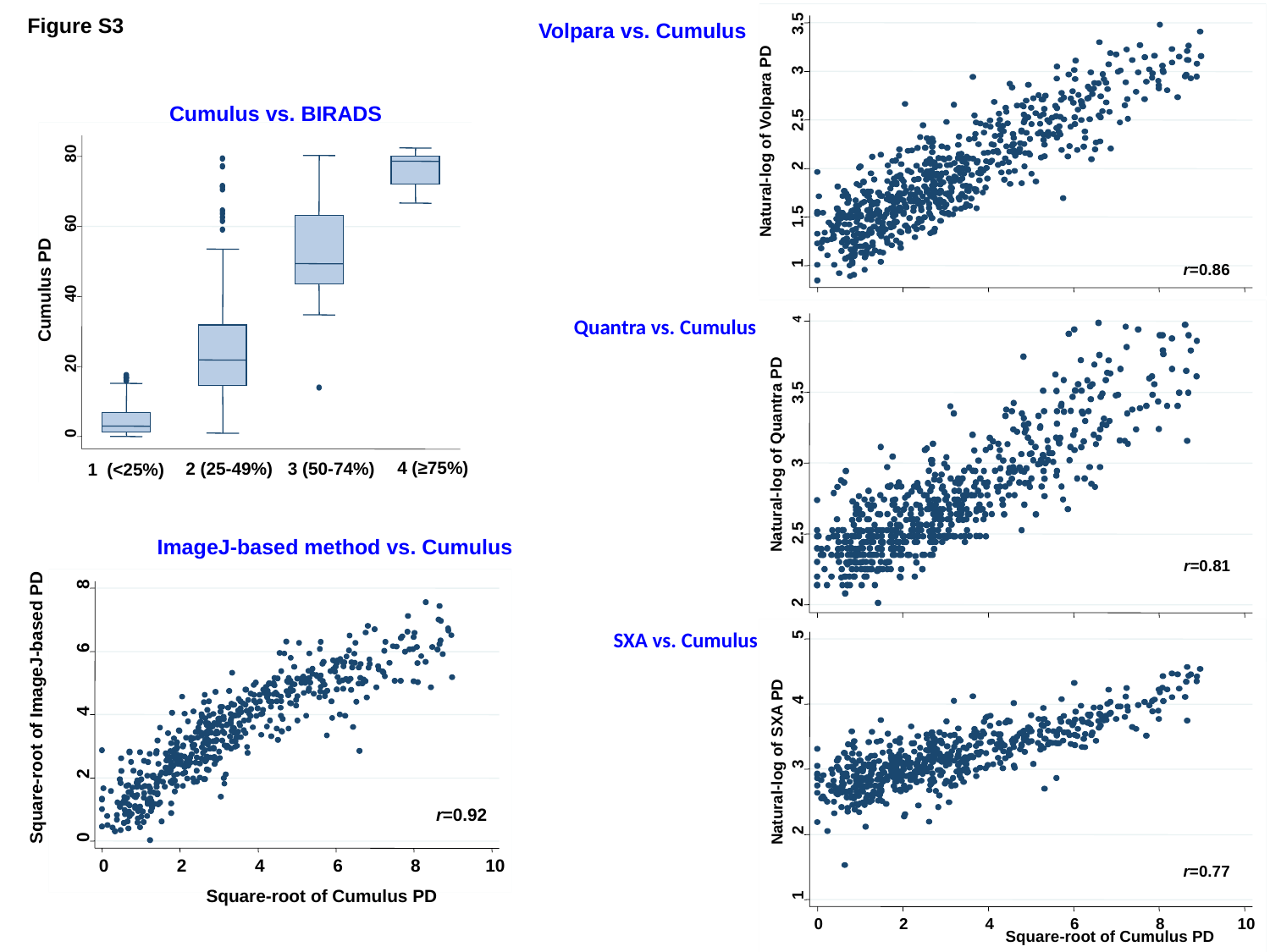

3.5
3
2.5
Natural-log of Volpara PD
2
1.5
1
r=0.86
0
2
4
6
8
10
Cumulus PD
Figure S3
Volpara vs. Cumulus
 Cumulus vs. BIRADS
80
60
Cumulus PD
40
20
0
1
2
3
4
4 (≥75%)
2 (25-49%)
3 (50-74%)
1 (<25%)
4
3.5
Natural-log of Quantra PD
3
2.5
r=0.81
2
0
2
4
6
8
10
Cumulus PD
Quantra vs. Cumulus
ImageJ-based method vs. Cumulus
8
6
Square-root of ImageJ-based PD
4
2
r=0.92
0
0
2
4
6
8
10
Square-root of Cumulus PD
5
4
Natural-log of SXA PD
3
2
r=0.77
1
0
2
4
6
8
10
Square-root of Cumulus PD
SXA vs. Cumulus

## Slide 4
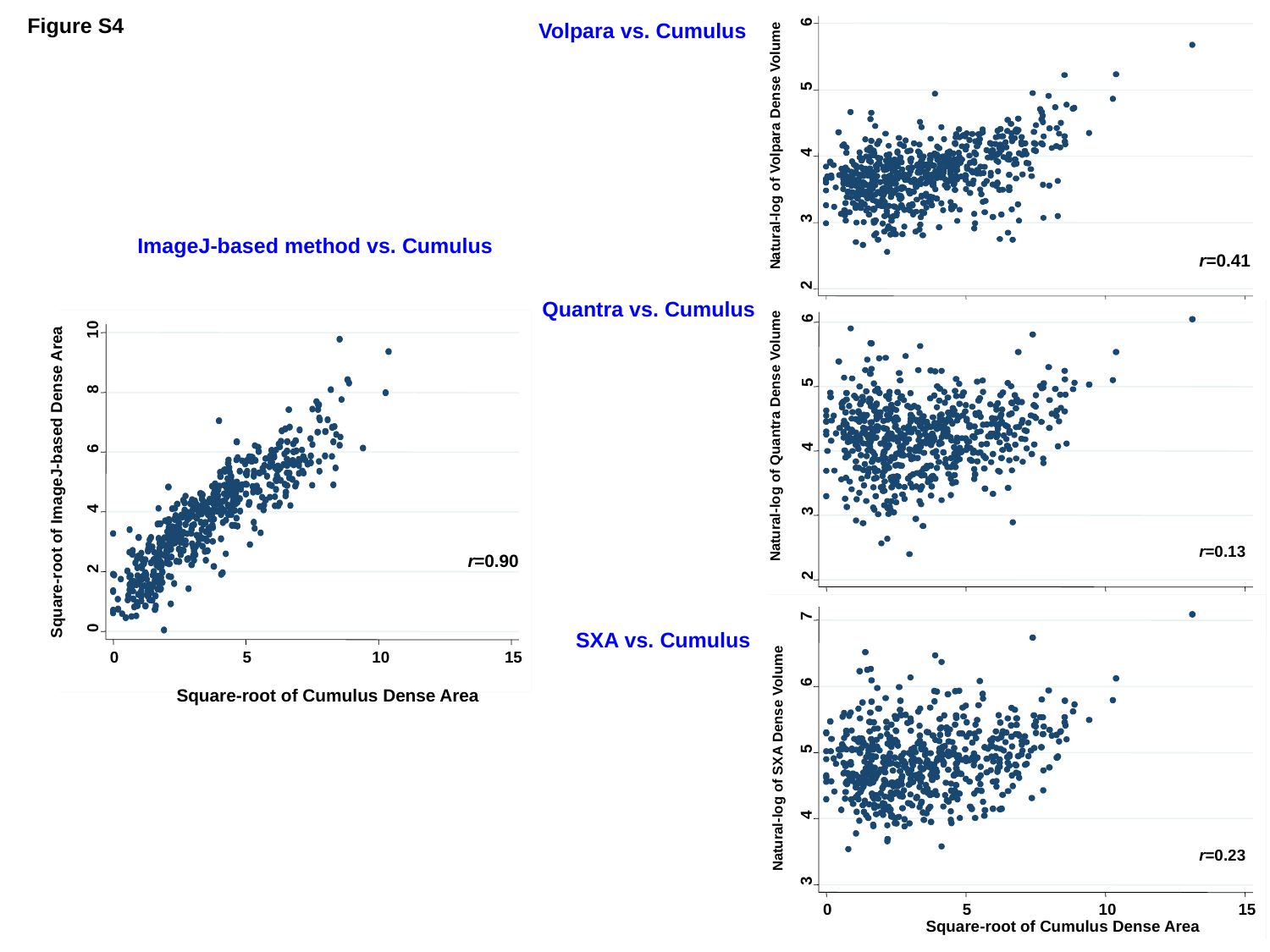

6
5
Natural-log of Volpara Dense Volume
4
3
r=0.41
2
0
5
10
15
Cumulus Dense Area
Figure S4
Volpara vs. Cumulus
ImageJ-based method vs. Cumulus
Quantra vs. Cumulus
6
5
Natural-log of Quantra Dense Volume
4
3
r=0.13
2
0
5
10
15
Cumulus Dense Area
10
8
6
Square-root of ImageJ-based Dense Area
4
r=0.90
2
0
0
5
10
15
Square-root of Cumulus Dense Area
7
6
5
Natural-log of SXA Dense Volume
4
r=0.23
3
0
5
10
15
Square-root of Cumulus Dense Area
SXA vs. Cumulus

## Slide 5
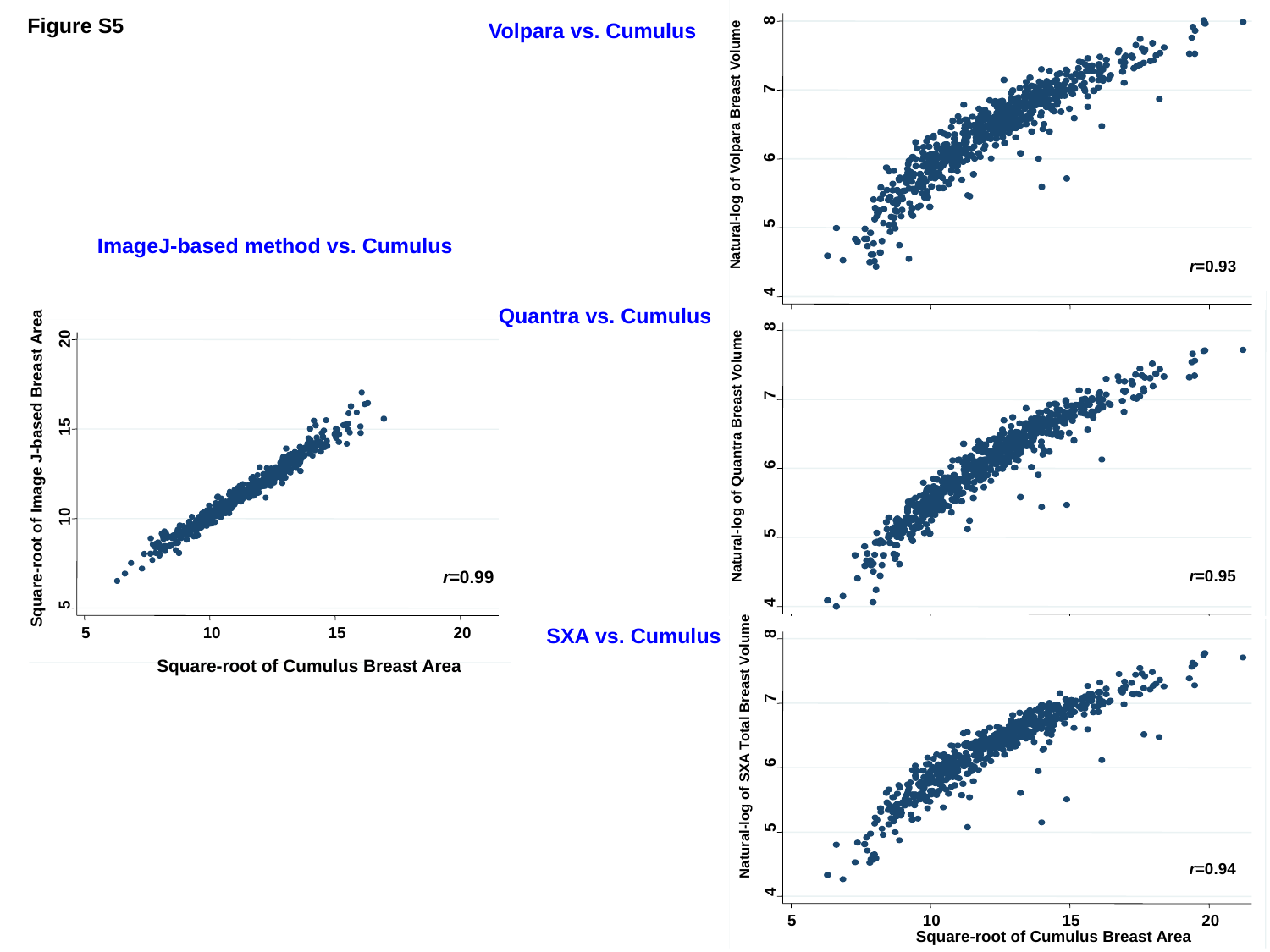

8
7
Natural-log of Volpara Breast Volume
6
5
r=0.93
4
5
10
15
20
Cumulus Total Breast Area
Figure S5
Volpara vs. Cumulus
ImageJ-based method vs. Cumulus
Quantra vs. Cumulus
8
7
Natural-log of Quantra Breast Volume
6
5
r=0.95
4
5
10
15
20
Cumulus Total Breast Area
20
15
Square-root of Image J-based Breast Area
10
r=0.99
5
5
10
15
20
Square-root of Cumulus Breast Area
8
7
Natural-log of SXA Total Breast Volume
6
5
r=0.94
4
5
10
15
20
Square-root of Cumulus Breast Area
SXA vs. Cumulus

## Slide 6
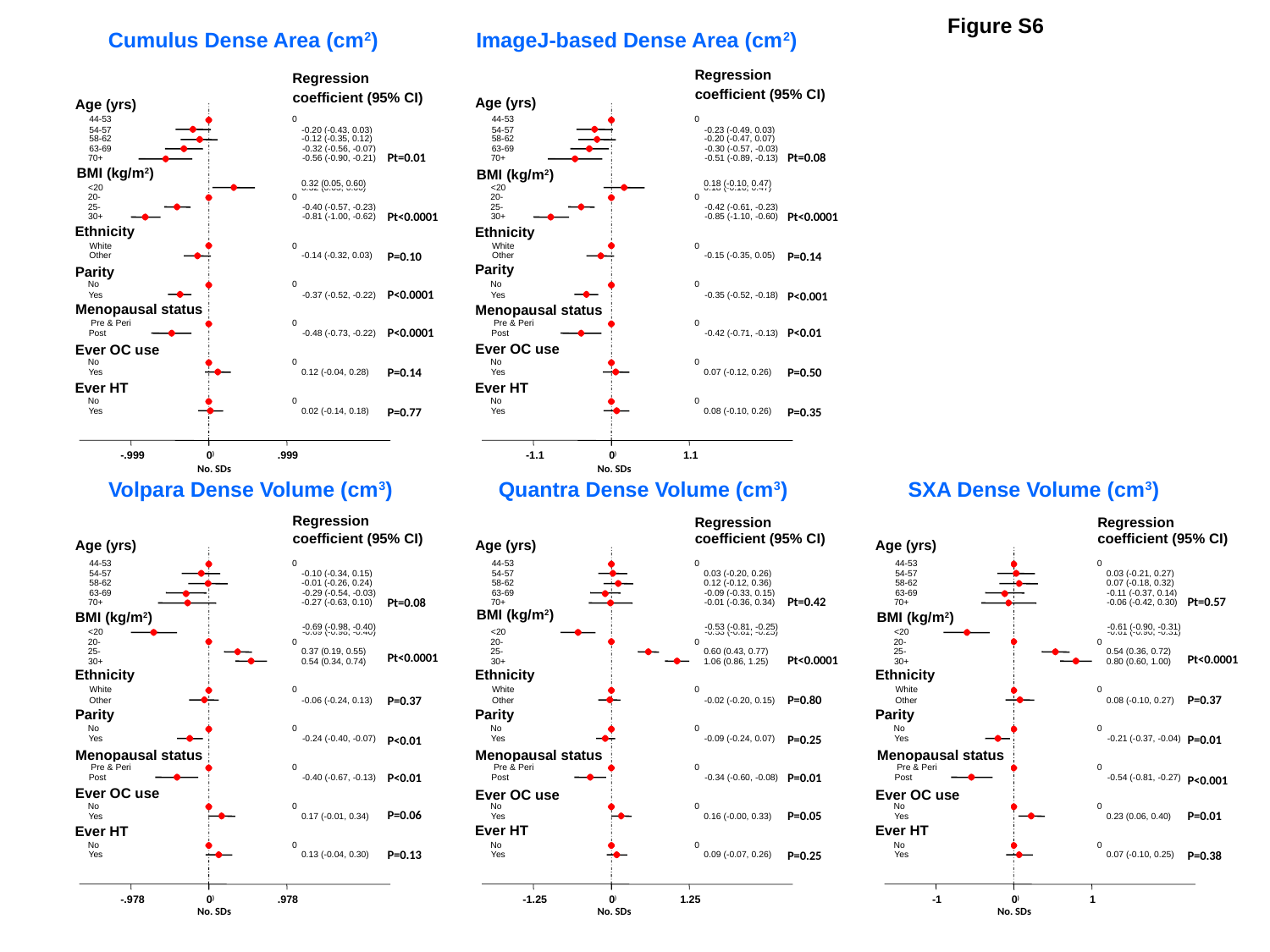

Cumulus Dense Area (cm2)
Regression
Regression
Coefficient (95% CI)
coefficient (95% CI)
Age (yrs)
Age (yrs)
44-53
0
44-53
0
54-57
-0.20 (-0.43, 0.03)
-0.20 (-0.43, 0.03)
54-57
-0.23 (-0.49, 0.03)
58-62
-0.12 (-0.35, 0.12)
-0.12 (-0.35, 0.12)
58-62
-0.20 (-0.47, 0.07)
63-69
-0.32 (-0.56, -0.07)
-0.32 (-0.56, -0.07)
63-69
-0.30 (-0.57, -0.03)
70+
-0.56 (-0.90, -0.21)
-0.56 (-0.90, -0.21)
70+
-0.51 (-0.89, -0.13)
BMI (kg/m2)
BMI (kg/m2)
0.32 (0.05, 0.60)
<20
0.32 (0.05, 0.60)
<20
0.18 (-0.10, 0.47)
20-
0
20-
0
25-
-0.40 (-0.57, -0.23)
-0.40 (-0.57, -0.23)
25-
-0.42 (-0.61, -0.23)
30+
-0.81 (-1.00, -0.62)
-0.81 (-1.00, -0.62)
30+
-0.85 (-1.10, -0.60)
Ethnicity
Ethnicity
White
0
White
0
Other
-0.14 (-0.32, 0.03)
-0.14 (-0.32, 0.03)
Other
-0.15 (-0.35, 0.05)
Parity
Parity
No
0
No
0
Yes
-0.37 (-0.52, -0.22)
-0.37 (-0.52, -0.22)
Yes
-0.35 (-0.52, -0.18)
Menopausal status
Menopausal status
Pre & Peri
0
Pre & Peri
0
Post
-0.48 (-0.73, -0.22)
-0.48 (-0.73, -0.22)
Post
-0.42 (-0.71, -0.13)
Ever OC use
Ever OC use
No
0
No
0
Yes
0.12 (-0.04, 0.28)
0.12 (-0.04, 0.28)
Yes
0.07 (-0.12, 0.26)
Ever HT
Ever HT
No
0
No
0
Yes
0.02 (-0.14, 0.18)
0.02 (-0.14, 0.18)
Yes
0.08 (-0.10, 0.26)
-.999
0
0
.999
ImageJ-based Dense Area (cm2)
coefficient (95% CI)
-0.23 (-0.49, 0.03)
-0.20 (-0.47, 0.07)
-0.30 (-0.57, -0.03)
-0.51 (-0.89, -0.13)
0.18 (-0.10, 0.47)
-0.42 (-0.61, -0.23)
-0.85 (-1.10, -0.60)
-0.15 (-0.35, 0.05)
-0.35 (-0.52, -0.18)
-0.42 (-0.71, -0.13)
0.07 (-0.12, 0.26)
0.08 (-0.10, 0.26)
-1.1
0
0
1.1
Volpara Dense Volume (cm3)
Regression
coefficient (95% CI)
Age (yrs)
44-53
0
54-57
-0.10 (-0.34, 0.15)
-0.10 (-0.34, 0.15)
58-62
-0.01 (-0.26, 0.24)
-0.01 (-0.26, 0.24)
63-69
-0.29 (-0.54, -0.03)
-0.29 (-0.54, -0.03)
70+
-0.27 (-0.63, 0.10)
-0.27 (-0.63, 0.10)
BMI (kg/m2)
-0.69 (-0.98, -0.40)
<20
-0.69 (-0.98, -0.40)
20-
0
25-
0.37 (0.19, 0.55)
0.37 (0.19, 0.55)
30+
0.54 (0.34, 0.74)
0.54 (0.34, 0.74)
Ethnicity
White
0
Other
-0.06 (-0.24, 0.13)
-0.06 (-0.24, 0.13)
Parity
No
0
Yes
-0.24 (-0.40, -0.07)
-0.24 (-0.40, -0.07)
Menopausal status
Pre & Peri
0
Post
-0.40 (-0.67, -0.13)
-0.40 (-0.67, -0.13)
Ever OC use
No
0
Yes
0.17 (-0.01, 0.34)
0.17 (-0.01, 0.34)
Ever HT
No
0
Yes
0.13 (-0.04, 0.30)
0.13 (-0.04, 0.30)
-.978
0
0
.978
Quantra Dense Volume (cm3)
Regression
Regression
coefficient (95% CI)
coefficient (95% CI)
Age (yrs)
Age (yrs)
44-53
0
44-53
0
54-57
0.03 (-0.20, 0.26)
0.03 (-0.20, 0.26)
54-57
0.03 (-0.21, 0.27)
0.03 (-0.21, 0.27)
58-62
0.12 (-0.12, 0.36)
0.12 (-0.12, 0.36)
58-62
0.07 (-0.18, 0.32)
0.07 (-0.18, 0.32)
63-69
-0.09 (-0.33, 0.15)
-0.09 (-0.33, 0.15)
63-69
-0.11 (-0.37, 0.14)
-0.11 (-0.37, 0.14)
70+
-0.01 (-0.36, 0.34)
-0.01 (-0.36, 0.34)
70+
-0.06 (-0.42, 0.30)
-0.06 (-0.42, 0.30)
BMI (kg/m2)
BMI (kg/m2)
-0.53 (-0.81, -0.25)
-0.61 (-0.90, -0.31)
<20
-0.53 (-0.81, -0.25)
<20
-0.61 (-0.90, -0.31)
20-
0
20-
0
25-
0.60 (0.43, 0.77)
0.60 (0.43, 0.77)
25-
0.54 (0.36, 0.72)
0.54 (0.36, 0.72)
30+
1.06 (0.86, 1.25)
1.06 (0.86, 1.25)
30+
0.80 (0.60, 1.00)
0.80 (0.60, 1.00)
Ethnicity
Ethnicity
White
0
White
0
Other
-0.02 (-0.20, 0.15)
-0.02 (-0.20, 0.15)
Other
0.08 (-0.10, 0.27)
0.08 (-0.10, 0.27)
Parity
Parity
No
0
No
0
Yes
-0.09 (-0.24, 0.07)
-0.09 (-0.24, 0.07)
Yes
-0.21 (-0.37, -0.04)
-0.21 (-0.37, -0.04)
Menopausal status
Menopausal status
Pre & Peri
0
Pre & Peri
0
Post
-0.34 (-0.60, -0.08)
-0.34 (-0.60, -0.08)
Post
-0.54 (-0.81, -0.27)
-0.54 (-0.81, -0.27)
Ever OC use
Ever OC use
No
0
No
0
Yes
0.16 (-0.00, 0.33)
0.16 (-0.00, 0.33)
Yes
0.23 (0.06, 0.40)
0.23 (0.06, 0.40)
Ever HT
Ever HT
No
0
No
0
Yes
0.09 (-0.07, 0.26)
0.09 (-0.07, 0.26)
Yes
0.07 (-0.10, 0.25)
0.07 (-0.10, 0.25)
-1.25
0
0
1.25
SXA Dense Volume (cm3)
-1
0
0
1
Figure S6
Pt=0.08
Pt=0.01
Pt<0.0001
Pt<0.0001
P=0.14
P=0.10
P<0.0001
P<0.001
P<0.0001
P<0.01
P=0.14
P=0.50
P=0.35
P=0.77
No. SDs
No. SDs
Pt=0.42
Pt=0.57
Pt=0.08
Pt<0.0001
Pt<0.0001
Pt<0.0001
P=0.80
P=0.37
P=0.37
P=0.25
P=0.01
P<0.01
P<0.01
P=0.01
P<0.001
P=0.06
P=0.05
P=0.01
P=0.13
P=0.38
P=0.25
No. SDs
No. SDs
No. SDs

## Slide 7
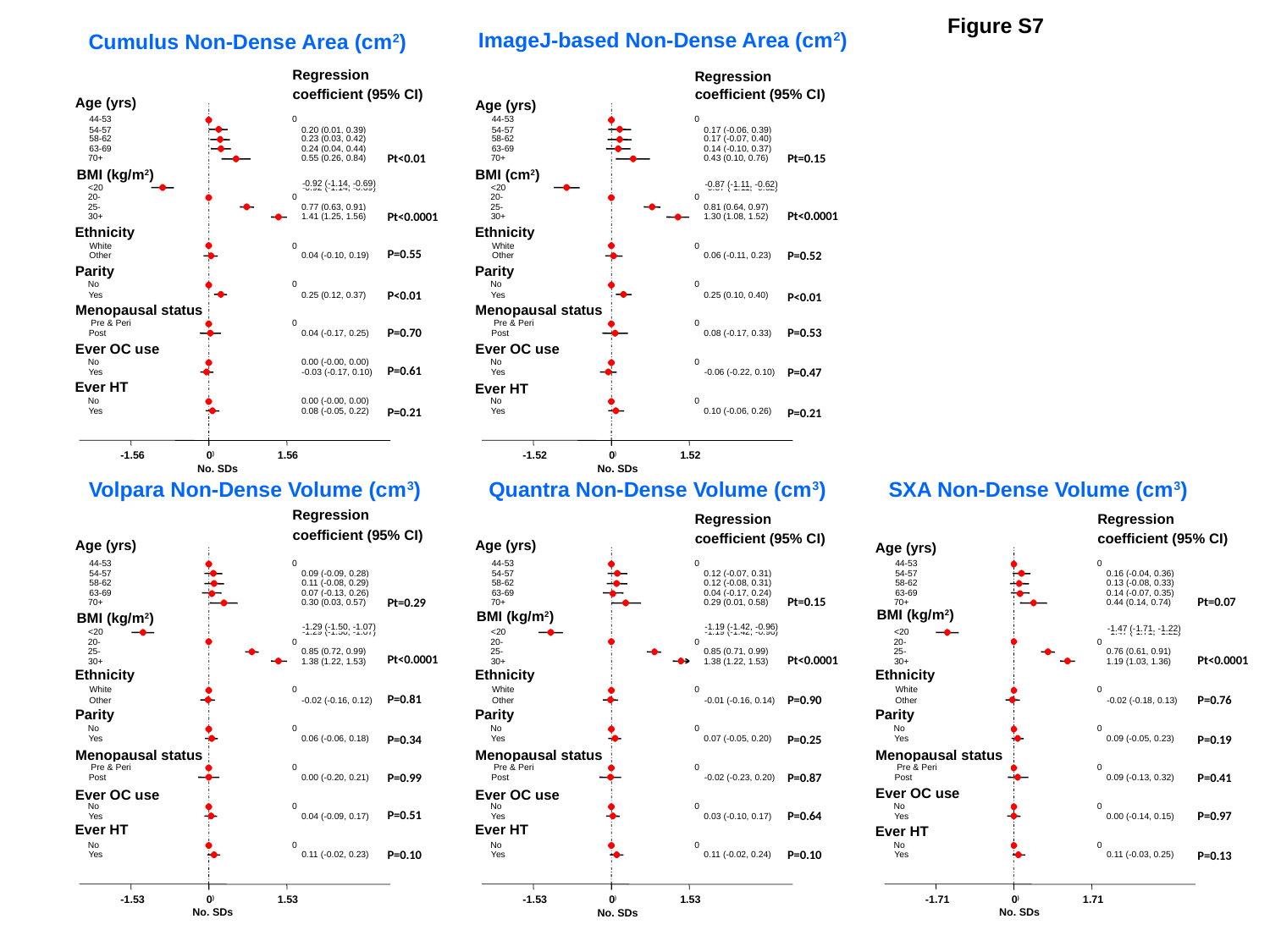

Cumulus Non-Dense Area (cm2)
Regression
Regression
coefficient (95% CI)
coefficient (95% CI)
Age (yrs)
Age (yrs)
44-53
0
44-53
0
54-57
0.20 (0.01, 0.39)
0.20 (0.01, 0.39)
54-57
0.17 (-0.06, 0.39)
58-62
0.23 (0.03, 0.42)
0.23 (0.03, 0.42)
58-62
0.17 (-0.07, 0.40)
63-69
0.24 (0.04, 0.44)
0.24 (0.04, 0.44)
63-69
0.14 (-0.10, 0.37)
70+
0.55 (0.26, 0.84)
0.55 (0.26, 0.84)
70+
0.43 (0.10, 0.76)
BMI (kg/m2)
BMI (cm2)
-0.92 (-1.14, -0.69)
<20
-0.92 (-1.14, -0.69)
<20
-0.87 (-1.11, -0.62)
20-
0
20-
0
25-
0.77 (0.63, 0.91)
0.77 (0.63, 0.91)
25-
0.81 (0.64, 0.97)
30+
1.41 (1.25, 1.56)
1.41 (1.25, 1.56)
30+
1.30 (1.08, 1.52)
Ethnicity
Ethnicity
White
0
White
0
Other
0.04 (-0.10, 0.19)
0.04 (-0.10, 0.19)
Other
0.06 (-0.11, 0.23)
Parity
Parity
No
0
No
0
Yes
0.25 (0.12, 0.37)
0.25 (0.12, 0.37)
Yes
0.25 (0.10, 0.40)
Menopausal status
Menopausal status
Pre & Peri
0
Pre & Peri
0
Post
0.04 (-0.17, 0.25)
0.04 (-0.17, 0.25)
Post
0.08 (-0.17, 0.33)
Ever OC use
Ever OC use
No
0.00 (-0.00, 0.00)
0.00 (-0.00, 0.00)
No
0
Yes
-0.03 (-0.17, 0.10)
-0.03 (-0.17, 0.10)
Yes
-0.06 (-0.22, 0.10)
Ever HT
Ever HT
No
0.00 (-0.00, 0.00)
0.00 (-0.00, 0.00)
No
0
Yes
0.08 (-0.05, 0.22)
0.08 (-0.05, 0.22)
Yes
0.10 (-0.06, 0.26)
-1.56
0
0
1.56
ImageJ-based Non-Dense Area (cm2)
0.17 (-0.06, 0.39)
0.17 (-0.07, 0.40)
0.14 (-0.10, 0.37)
0.43 (0.10, 0.76)
-0.87 (-1.11, -0.62)
0.81 (0.64, 0.97)
1.30 (1.08, 1.52)
0.06 (-0.11, 0.23)
0.25 (0.10, 0.40)
0.08 (-0.17, 0.33)
-0.06 (-0.22, 0.10)
0.10 (-0.06, 0.26)
-1.52
0
0
1.52
Volpara Non-Dense Volume (cm3)
Regression
coefficient (95% CI)
Age (yrs)
44-53
0
54-57
0.09 (-0.09, 0.28)
0.09 (-0.09, 0.28)
58-62
0.11 (-0.08, 0.29)
0.11 (-0.08, 0.29)
63-69
0.07 (-0.13, 0.26)
0.07 (-0.13, 0.26)
70+
0.30 (0.03, 0.57)
0.30 (0.03, 0.57)
BMI (kg/m2)
-1.29 (-1.50, -1.07)
<20
-1.29 (-1.50, -1.07)
20-
0
25-
0.85 (0.72, 0.99)
0.85 (0.72, 0.99)
30+
1.38 (1.22, 1.53)
1.38 (1.22, 1.53)
Ethnicity
White
0
Other
-0.02 (-0.16, 0.12)
-0.02 (-0.16, 0.12)
Parity
No
0
Yes
0.06 (-0.06, 0.18)
0.06 (-0.06, 0.18)
Menopausal status
Pre & Peri
0
Post
0.00 (-0.20, 0.21)
0.00 (-0.20, 0.21)
Ever OC use
No
0
Yes
0.04 (-0.09, 0.17)
0.04 (-0.09, 0.17)
Ever HT
No
0
Yes
0.11 (-0.02, 0.23)
0.11 (-0.02, 0.23)
-1.53
0
0
1.53
Quantra Non-Dense Volume (cm3)
Regression
Regression
coefficient (95% CI)
coefficient (95% CI)
Age (yrs)
Age (yrs)
44-53
0
44-53
0
54-57
0.12 (-0.07, 0.31)
0.12 (-0.07, 0.31)
54-57
0.16 (-0.04, 0.36)
0.16 (-0.04, 0.36)
58-62
0.12 (-0.08, 0.31)
0.12 (-0.08, 0.31)
58-62
0.13 (-0.08, 0.33)
0.13 (-0.08, 0.33)
63-69
0.04 (-0.17, 0.24)
0.04 (-0.17, 0.24)
63-69
0.14 (-0.07, 0.35)
0.14 (-0.07, 0.35)
70+
0.29 (0.01, 0.58)
0.29 (0.01, 0.58)
70+
0.44 (0.14, 0.74)
0.44 (0.14, 0.74)
BMI (kg/m2)
BMI (kg/m2)
-1.19 (-1.42, -0.96)
-1.47 (-1.71, -1.22)
<20
-1.19 (-1.42, -0.96)
<20
-1.47 (-1.71, -1.22)
20-
0
20-
0
25-
0.85 (0.71, 0.99)
0.85 (0.71, 0.99)
25-
0.76 (0.61, 0.91)
0.76 (0.61, 0.91)
30+
1.38 (1.22, 1.53)
1.38 (1.22, 1.53)
30+
1.19 (1.03, 1.36)
1.19 (1.03, 1.36)
Ethnicity
Ethnicity
White
0
White
0
Other
-0.01 (-0.16, 0.14)
-0.01 (-0.16, 0.14)
Other
-0.02 (-0.18, 0.13)
-0.02 (-0.18, 0.13)
Parity
Parity
No
0
No
0
Yes
0.07 (-0.05, 0.20)
0.07 (-0.05, 0.20)
Yes
0.09 (-0.05, 0.23)
0.09 (-0.05, 0.23)
Menopausal status
Menopausal status
Pre & Peri
0
Pre & Peri
0
Post
-0.02 (-0.23, 0.20)
-0.02 (-0.23, 0.20)
Post
0.09 (-0.13, 0.32)
0.09 (-0.13, 0.32)
Ever OC use
Ever OC use
No
0
No
0
Yes
0.03 (-0.10, 0.17)
0.03 (-0.10, 0.17)
Yes
0.00 (-0.14, 0.15)
0.00 (-0.14, 0.15)
Ever HT
Ever HT
No
0
No
0
Yes
0.11 (-0.02, 0.24)
0.11 (-0.02, 0.24)
Yes
0.11 (-0.03, 0.25)
0.11 (-0.03, 0.25)
-1.53
0
0
1.53
SXA Non-Dense Volume (cm3)
-1.71
0
0
1.71
Figure S7
Pt<0.01
Pt=0.15
Pt<0.0001
Pt<0.0001
P=0.55
P=0.52
P<0.01
P<0.01
P=0.70
P=0.53
P=0.61
P=0.47
P=0.21
P=0.21
No. SDs
No. SDs
Pt=0.15
Pt=0.07
Pt=0.29
Pt<0.0001
Pt<0.0001
Pt<0.0001
P=0.81
P=0.90
P=0.76
P=0.34
P=0.25
P=0.19
P=0.41
P=0.99
P=0.87
P=0.51
P=0.64
P=0.97
P=0.10
P=0.10
P=0.13
No. SDs
No. SDs
No. SDs
