# Supplementary material for: Digital mammographic density and breast cancer risk: a case–control study of six alternative density assessment methods
Source: Breast Cancer Res. 2014 Sep 20;16:439. doi: 10.1186/s13058-014-0439-1 (PMC4303120; doi:10.1186/s13058-014-0439-1)
Supplement: Supplementary file 3 — Additional file 3: Table S1.: Spearman's rank correlation coefficients (r) between the various quantitative methods of quantifying percent density in control women. Table S2. Quintile agreement between the five quantitative methods of quantifying percent density in control women. Table S3. Distribution of density readings and level of agreement by method, breast and view in control women. Table S4. Area under the receiving operating curve (AUC) for percent and absolute density for each quantitative method. (DOCX 47 KB) [file 13058_2014_439_MOESM3_ESM.docx]

**Table S1**. Spearman’s rank correlation coefficients (*r*) between the various quantitative methods of quantifying percent density in control women ^a^

|  | **Area-based**  **methods** | | **Volumetric**  **methods** | | |
| --- | --- | --- | --- | --- | --- |
|  | **Cumulus** | **Image J** | **Volpara** | **Quantra** | **SXA** |
| **Cumulus** | - | 0.92 | 0.86 | 0.81 | 0.77 |
| **Image J** |  | - | 0.87 | 0.78 | 0.79 |
| **Volpara** |  |  | - | 0.91 | 0.76 |
| **Quantra** |  |  |  | - | 0.77 |
| **SXA** |  |  |  |  | - |

^a^ Based on the mean measurements for each woman (4 images per woman for

Cumulus, ImageJ and Volpara; 2 for Quantra and SXA) (log transformed for

volumetric methods and square root transformed for area-based methods)

**Table S2.** Quintile agreement between the five quantitative methods of quantifying percent density in control women (n=466 ^a^)

|  | **Area-based methods ^b^** | | | | **Volumetric methods ^b^** | | | | | |
| --- | --- | --- | --- | --- | --- | --- | --- | --- | --- | --- |
|  | **Cumulus** | | **ImageJ** | | **Volpara** | | **Quantra** | | **SXA** | |
|  | **Same Q** | **Same ±1 Q** | **Same Q** | **Same ±1 Q** | **Same Q** | **Same ±1 Q** | **Same Q** | **Same ±1 Q** | **Same Q** | **Same ±1 Q** |
|  |  |  |  |  |  |  |  |  |  |  |
| **Cumulus** | - | - | 290 *(62%)* | 453 *(97%)* | 258 *(55%)* | 432 *(93%)* | 241 (52%) | 424 *(91%)* | 223 *(48%)* | 414 *(89%)* |
| **ImageJ** | 290 *(62%)* | 453 *(97%)* | - | - | 233 *(50%)* | 427 *(92%)* | 219 (47%) | 406 *(87%)* | 228 *(49%)* | 407 *(87%)* |
| **Volpara** | 258 *(55%)* | 432 *(93%)* | 233 *(50%)* | 427 *(92%)* | - | - | 309 (66%) | 451 *(97%)* | 235 *(50%)* | 425 *(91%)* |
| **Quantra** | 241 *(52%)* | 424 *(91%)* | 219 *(47%)* | 406 *(87%)* | 309 *(66%)* | 451 *(97%)* | - | - | 235 *(50%)* | 421 *(90%)* |
| **SXA** | 223 *(48%)* | 414 *(89%)* | 228 *(49%)* | 407 *(87%)* | 235 *(50%)* | 425 *(91%)* | 235 (50%) | 421 *(90%)* | - | - |

^a^ Analyses restricted to the subset of women with percent density measurements from all five quantitative methods and based on average of all available readings (four images per woman for Cumulus, ImageJ-based method and Volpara; two images for Quantra and SXA – see Figure 1).

^b^ Numbers (%) of control women classified in the same quintile (Q) and in the same +1 adjacent quintiles.

|  | **AREA-BASED METHODS** | | | | | |
| --- | --- | --- | --- | --- | --- | --- |
|  | **CUMULUS** | | | **IMAGE J-BASED METHOD** | | |
|  | **Percent Density (%)** | **Dense Area (cm^2^)** | **Total Breast Area (cm^2^)** | **Percent Density (%)** | **Dense Area (cm^2^)** | **Total Breast Area**  **(cm^2^)** |
| **CC views** |  |  |  |  |  |  |
| Percentiles: 50th (25th, 75th) |  |  |  |  |  |  |
| Left | **6.8 (2.0-19.3)** | **9.8 (3.1-22.9)** | **144.8 (101.1-186.3)** | **12.5 (5.1-21.9)** | **14.4 (6.6-23.0)** | **128.0 (95.2-160.8)** |
| Right | **7.5 (2.3-21.7)** | **10.1 (3.2-25.1)** | **135.7 (96.9-179.3)** | **13.0 (5.6-23.6)** | **14.4 (6.9-24.1)** | **125.9 (92.2-158.0)** |
| Test for L-R difference ^a^ | **t=-4.52, P<0.001** | **t=-2.87, P<0.01** | **t=6.49, P<0.001** | **t=-3.05, P<0.01** | **t=-1.71, P=0.09** | **t=4.61, P<0.001** |
| Mean diff + Limits of agreement ^b^ | **-0.15 (-1.83,1.53)** | **-0.11 (-2.15,1.92)** | **0.16 (-1.09,1.40)** | **-0.13 (-2.04, 1.79** | **-0.08 (-2.20, 2.04)** | **0.15 (-1.36, 1.66)** |
| Left-Right Reliability |  |  |  |  |  |  |
| Single film | **0.92** | **0.89** | **0.97** | **0.84** | **0.80** | **0.93** |
| L-R mean | **0.96** | **0.94** | **0.98** | **0.91** | **0.89** | **0.96** |
| **MLO views** |  |  |  |  |  |  |
| Percentiles: 50th (25th, 75th) |  |  |  |  |  |  |
| Left | **6.5 (1.9-19.4)** | **10.3 (3.3-25.7)** | **158.2 (117.8-196.4)** | **11.3 (4.0-21.6)** | **15.0 (6.2-27.3)** | **141.5 (114.8-173.3)** |
| Right | **7.1 (1.7-20.9)** | **11.0 (3.1-27.9)** | **157.8 (118.1-200.3)** | **12.4 (4.2-23.4)** | **16.2 (6.4-28.0)** | **140.3 (112.5-170.2)** |
| Test for L-R difference ^a^ | **t=-1.57, P=0.12** | **t=-0.93, P=0.35** | **t=0.75, P=0.46** | **t=-2.65, P=0.01** | **t=-2.13, P=0.03** | **t=2.11, P=0.04** |
| Mean diff + Limits of agreement | **-0.05 (-1.63,1.53)** | **-0.04 (-2.04,1.96)** | **0.02 (-1.03, 1.06)** | **-0.11 (-1.96, 1.74)** | **-0.10 (-2.35, 2.14)** | **0.06 (-1.31, 1.44)** |
| Left-Right Reliability |  |  |  |  |  |  |
| Single film | **0.93** | **0.90** | **0.97** | **0.85** | **0.83** | **0.92** |
| L-R mean | **0.96** | **0.95** | **0.99** | **0.92** | **0.90** | **0.96** |
| ^a^  Log transformed for Volpara, Quantra and SXA; square root transformed for Cumulus and Image J | | | |  |  |  |
| ^b^ Limits of agreement refer to the range in which 95% of left-right differences are expected to lie, calculated as mean difference + 1.96SD (difference) | | | | | | |

**Table S3.** Distribution of mammographic density measures and level of agreement by method, breast and view in control women

**Table S3 (cont.)** Distribution percentiles, left-right breast agreement and reliability of the quantitative methods in control women

|  | **VOLUMETRIC METHODS** | | | | | | | | |
| --- | --- | --- | --- | --- | --- | --- | --- | --- | --- |
|  | **VOLPARA** | | | **QUANTRA** | | | **SXA** | | |
|  |  | | | Average of CC & MLO views | | | Only CC view available | | |
|  | Percent Density (%) | Dense Volume (cm^3^) | Total Breast Volume (cm^3^) | Percent Density (%) | Dense Volume (cm^3^) | Total Breast Volume (cm^3^) | Percent Density (%) | Dense Volume (cm^3^) | Total Breast Volume (cm^3^) |
| **CC views** |  |  |  |  |  |  |  |  |  |
| Percentiles: 50th (25th, 75th) |  |  |  |  |  |  |  |  |  |
| Left | **6.5 (4.6-10.1)** | **38.9 (28.5-51.9)** | **603.3 (362.7-873.7)** | **14 (12-19)** | **71 (51-96)** | **479 (280-697)** | **22.8 (17.2-31.9)** | **127.2 (90.8-182.2)** | **605.8 (372.3-840)** |
| Right | **6.6 (4.7-10.2)** | **37.6 (27.9-52.0)** | **581.3 (345.8-830.1)** | **15 (12-20)** | **70 (50-98)** | **461 (269-691)** | **24.0 (18.3-33.6)** | **128.2 (93.1-185.1)** | **571.1 (356.3-799)** |
| Test for L-R difference ^a^ | **t=-2.12, P=0.03** | **t=1.27, P=0.21** | **t=6.08, P<0.001** | **t=-6.16, P<0.001** | **t=-2.27, P=0.02** | **t=2.95, P<0.01** | **t=-6.66, P<0.001** | **t=-2.17, P=0.03** | **t=6.43, P<0.001** |
| Mean diff + Limits of agreement ^b^ | **-0.02 (-0.51,0.47)** | **0.01 (-0.53,0.56)** | **0.03 (-0.25,0.32)** | **-0.04 (-0.32,0.25)** | **-0.02 (-0.45,0.41)** | **0.02 (-0.27,0.31)** | **-0.06 (-0.47,0.36)** | **-0.02 (-0.48,0.44)** | **0.04 (-0.24, 0.31)** |
| Left-Right Reliability |  |  |  |  |  |  |  |  |  |
| Single film | **0.90** | **0.82** | **0.97** | **0.94** | **0.92** | **0.98** | **0.89** | **0.90** | **0.97** |
| L-R mean | **0.95** | **0.90** | **0.99** | **0.97** | **0.96** | **0.99** | **0.94** | **0.95** | **0.99** |
| **MLO views** |  |  |  |  |  |  |  |  |  |
| Percentiles: 50th (25th, 75th) |  |  |  |  |  |  |  |  |  |
| Left | **5.8 (4.4-9.1)** | **44.1 (33.5-58.0)** | **738.4 (448.1-1081.7)** |  |  |  |  |  |  |
| Right | **6.2 (4.6-10.1)** | **46.0 (34.5-60.8)** | **721.5 (431.8-1055.5)** |  |  |  |  |  |  |
| Test for L-R difference ^a^ | **t=-6.81, P<0.001** | **t=-3.34, P<0.001** | **t=4.94, P<0.001** |  |  |  |  |  |  |
| Mean diff + Limits of agreement ^b^ | **-0.06 (-0.51,0.39)** | **-0.03 (-0.54,0.48)** | **0.03 (-0.24,0.30)** |  |  |  |  |  |  |
| Left-Right Reliability |  |  |  |  |  |  |  |  |  |
| Single film | **0.90** | **0.82** | **0.98** |  |  |  |  |  |  |
| L-R mean | **0.95** | **0.90** | **0.99** |  |  |  |  |  |  |
| ^a^  Log transformed for Volpara, Quantra and SXA; square root transformed for Cumulus and Image J | | | |  |  |  |  |  |  |
| ^b^ Limits of agreement refer to the range in which 95% of left-right differences are expected to lie, calculated as mean difference + 1.96 standard deviation | | | | | | |  |  |  |

**Table S4**. Area under the receiving operating curve (AUC) for percent and absolute density for each quantitative method ^a^

| **Density measure:**  **Density assessment method** | **AUC ^b^** | **95% CI** |
| --- | --- | --- |
|  |  |  |
| **Percent density:** |  |  |
| **Area-based methods** |  |  |
| Cumulus | 0.65 | 0.60, 0.70 |
| ImageJ-based method | 0.68 | 0.63, 0.73 |
| **Volumetric methods** |  |  |
| Volpara | 0.65 | 0.60, 0.70 |
| Quantra | 0.63 | 0.58, 0.67 |
| SXA | 0.64 | 0.59, 0.69 |
|  |  |  |
| *P for difference between methods* | *P=0.005 ^c^* | |
|  |  |  |
| **Absolute density:** |  |  |
| **Area-based methods** |  |  |
| Cumulus | 0.66 | 0.61, 0.71 |
| ImageJ-based method | 0.69 | 0.64, 0.73 |
| **Volumetric methods** |  |  |
| Volpara | 0.67 | 0.62, 0.72 |
| Quantra | 0.63 | 0.58, 0.68 |
| SXA | 0.63 | 0.58, 0.73 |
|  |  |  |
| *P for difference between methods* | *P=0.008 ^d^* | |

^a^ Restricted to the subset of participants of screening ages (50-69 years) and with available measurements for all five quantitative methods.

^b^ Based on the average of CC and MLO view readings except for the SXA method which is based on the CC view only (see Methods). Models include age, BMI, parity and menopausal status.

^c^ Pair-wise comparisons show that the between-method differences in AUC were accounted for by borderline statistically significantly higher AUC for the ImageJ-based method (P=0.05) and lower AUC for Quantra (P=0.06) relative to Cumulus.

^d^ Pair-wise comparisons show that the between-method differences in AUC were accounted for by borderline statistically significantly higher AUC for the ImageJ-based method (P=0.06), and lower AUC for Quantra (P=0.04), both relative to Cumulus.
