# Supplementary figures and images for: Digital mammographic density and breast cancer risk: a case–control study of six alternative density assessment methods
Source: Breast Cancer Res. 2014 Sep 20;16:439. doi: 10.1186/s13058-014-0439-1 (PMC4303120; doi:10.1186/s13058-014-0439-1)

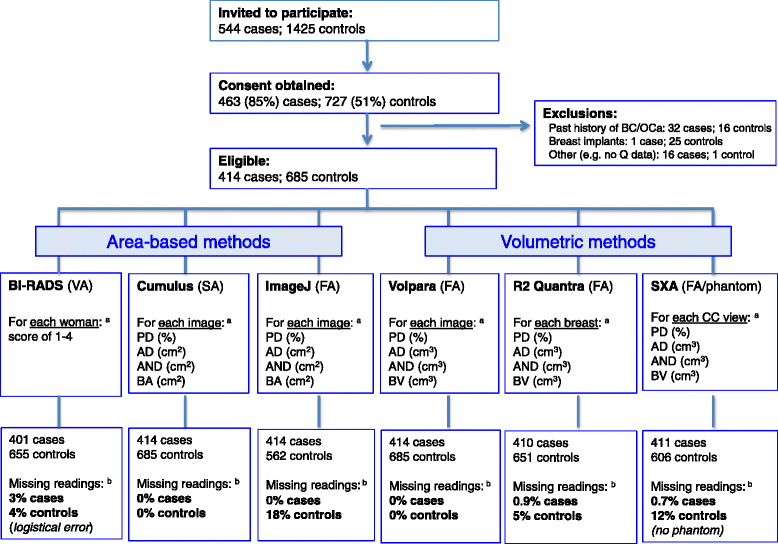

Supplement: Supplementary file 4 — Authors’ original file for figure 1 [file 13058_2014_439_MOESM4_ESM.gif]

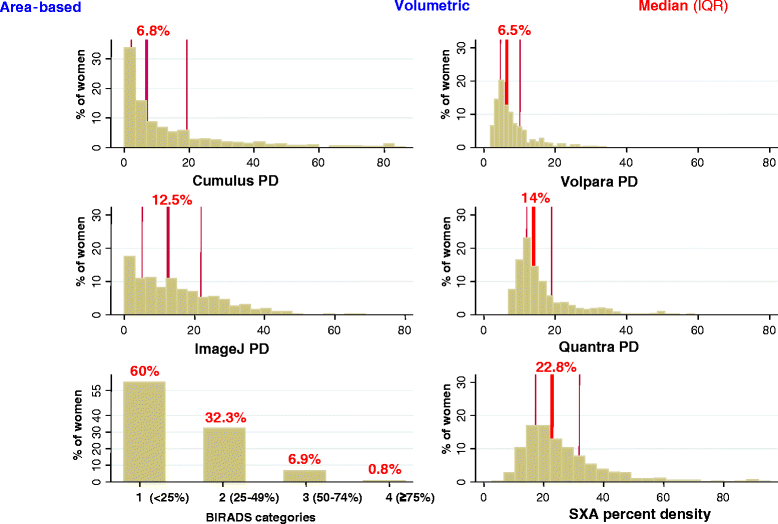

Supplement: Supplementary file 5 — Authors’ original file for figure 2 [file 13058_2014_439_MOESM5_ESM.gif]

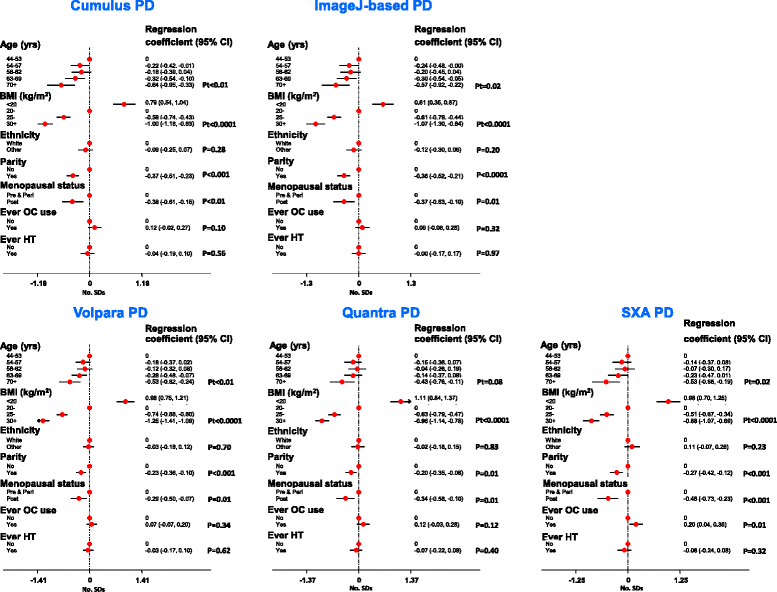

Supplement: Supplementary file 6 — Authors’ original file for figure 3 [file 13058_2014_439_MOESM6_ESM.gif]

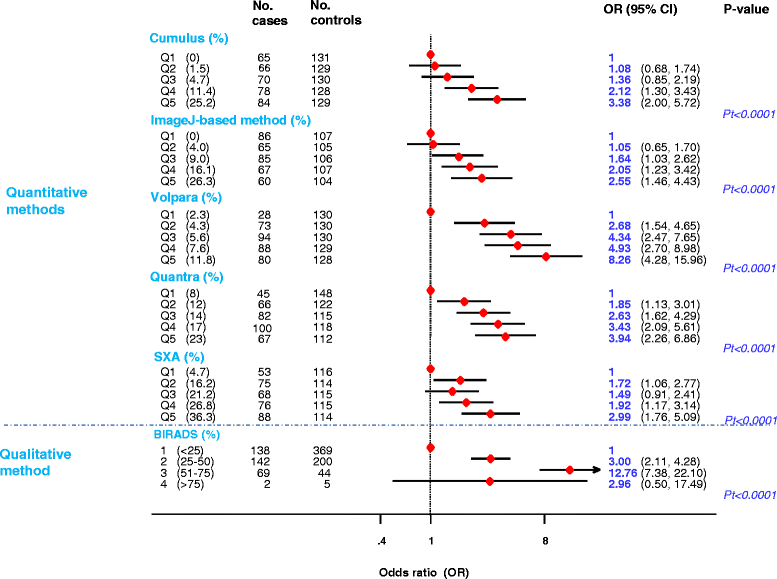

Supplement: Supplementary file 7 — Authors’ original file for figure 4 [file 13058_2014_439_MOESM7_ESM.gif]

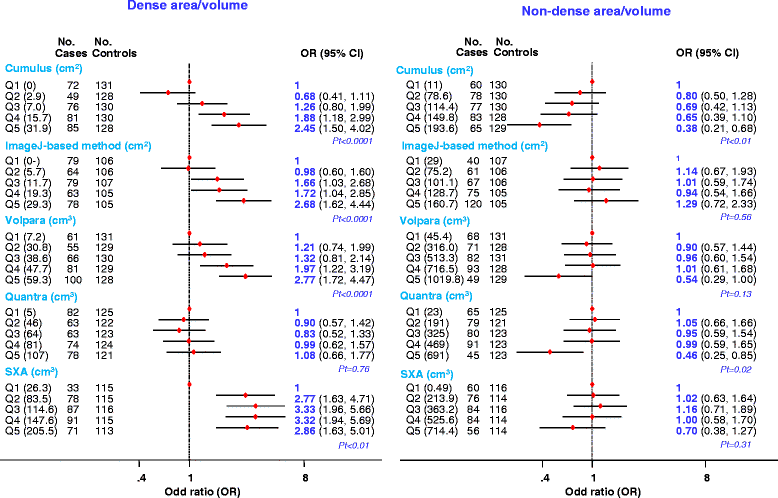

Supplement: Supplementary file 8 — Authors’ original file for figure 5 [file 13058_2014_439_MOESM8_ESM.gif]

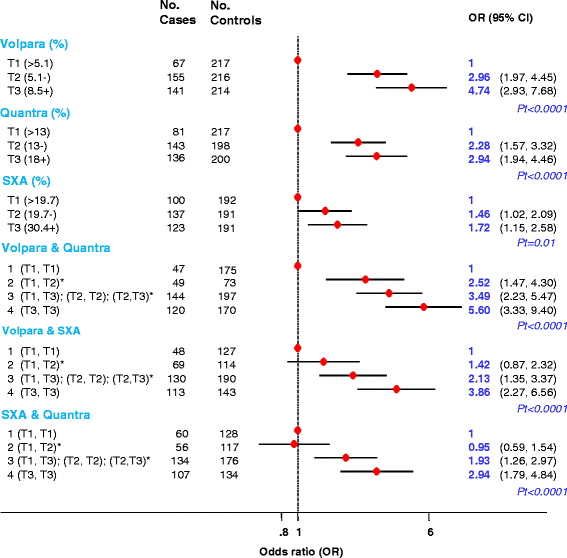

Supplement: Supplementary file 9 — Authors’ original file for figure 6 [file 13058_2014_439_MOESM9_ESM.gif]
